# Demonstration of CRISPR/Cas9/sgRNA-mediated targeted gene modification in Arabidopsis, tobacco, sorghum and rice

**DOI:** 10.1093/nar/gkt780

**Published:** 2013-08-31

**Authors:** Wenzhi Jiang, Huanbin Zhou, Honghao Bi, Michael Fromm, Bing Yang, Donald P. Weeks

**Affiliations:** ^1^Department of Biochemistry, University of Nebraska, Lincoln, NE 68588, USA, ^2^Deparment of Genetics, Development and Cell Biology, Iowa State University, Ames, IA 50011, USA and ^3^Department of Agronomy and Horticulture, University of Nebraska, Lincoln, NE 68588, USA

## Abstract

The type II CRISPR/Cas system from *Streptococcus pyogenes* and its simplified derivative, the Cas9/single guide RNA (sgRNA) system, have emerged as potent new tools for targeted gene knockout in bacteria, yeast, fruit fly, zebrafish and human cells. Here, we describe adaptations of these systems leading to successful expression of the Cas9/sgRNA system in two dicot plant species, Arabidopsis and tobacco, and two monocot crop species, rice and sorghum. *Agrobacterium tumefaciens* was used for delivery of genes encoding Cas9, sgRNA and a non-fuctional, mutant green fluorescence protein (GFP) to Arabidopsis and tobacco. The mutant GFP gene contained target sites in its 5′ coding regions that were successfully cleaved by a CAS9/sgRNA complex that, along with error-prone DNA repair, resulted in creation of functional GFP genes. DNA sequencing confirmed Cas9/sgRNA-mediated mutagenesis at the target site. Rice protoplast cells transformed with Cas9/sgRNA constructs targeting the promoter region of the bacterial blight susceptibility genes, *OsSWEET14* and *OsSWEET11*, were confirmed by DNA sequencing to contain mutated DNA sequences at the target sites. Successful demonstration of the Cas9/sgRNA system in model plant and crop species bodes well for its near-term use as a facile and powerful means of plant genetic engineering for scientific and agricultural applications.

## INTRODUCTION

The ability to target specific genes for knockout or replacement by homologous recombination has proved powerful in bacterial and yeast systems for rapid elucidation of metabolic pathways and regulatory mechanisms. In the 1980s, the advent of reliable (but costly and technically demanding) methods for gene knockout (and later, gene knockin) in mice opened fruitful avenues for better identifying and understanding mammalian genes and discovering their roles in maintaining cell health and viability (1–3). These discoveries have greatly aided elucidation of processes by which disease can arise and have allowed design and testing of molecular and genetic strategies for restoring health in diseased animals and humans (e.g. 4–9).

In recent years, new methods have been developed that extended targeted gene disruption technologies to human cells in culture, numerous other model animal systems (e.g. fruit flies, zebrafish, nematodes), algae and plants. The first of these, zinc finger nuclease (ZFN) technology (10), weds the non-specific nuclease domain of a subunit of the restriction enzyme, *FokI*, to a set of synthetic zinc finger proteins whose ability to recognize a specific DNA sequence allows targeting of a single gene even within a highly complex eukaryotic genome. A pair of ZFNs binding head to head on opposite strands of adjacent DNA sequences facilitates dimerization of the two attached *FokI* nuclease subunits and cleavage of the DNA sequence region separating the two ZFNs. Subsequent error-prone DNA repair by the nonhomologous end-joining (NHEJ) system often leads to small deletions or insertions of nucleotides that, in the coding region of a gene, can result in a shift in the codon reading frame and, thus, gene knockouts. If at the time of DNA cleavage by a pair of ZFNs, a piece of DNA with strong homology to the severed gene is present, the chances of gene replacement by homologous recombination increase significantly (11,12). ZFNs have proven highly valuable in defining the roles of numerous genes in cells from a variety of organisms, including humans, mice, farm animals, fruit flies, nematodes, fish, algae and higher plants [reviewed in (13)]. However, ZFN technology suffers from difficulties in design, construction, cost and uncertain success rates.

Within the past 3 years, TAL effector nuclease (TALEN) technology has come to the fore as a more tractable system for targeted gene disruption and, in some cases, gene replacement [reviewed in (13,14)]. Successful targeting of TALENs to a specific DNA sequence is possible due to the relatively simple ‘code’ (15,16) that matches a well-defined di-amino acid sequence [repeat-variable di-residue (RVD)] within a highly conserved repeat unit of ∼33–35 amino acid with one of the four nucleotides [e.g. an asparagine/asparagine (N/N) RVD pairs with a guanosine (G) nucleotide, an asparagine and glycine (N/G) RVD with T, a histidine and aspartic acid (H/D) RVD with a C and an asparagine and isoleucine (N/I) RVD with an A]. Because the four different TALEN repeat units can be artificially assembled in any desired order, TALENs, in theory, can target any DNA target within any gene in any organism. The development of a number of facile methods for rapid synthesis and assembly of TALENs [e.g. (17–20)] allows researchers to perform experiments targeting a single or several sites within a gene of interest. The power of this technology, while still emerging, is evident from the numerous genes in a large variety of organisms that have been disrupted, added, deleted or replaced using precisely targeted TALENs [e.g. knockout of the CCR5 gene for HIV resistance in human cells (21); disruption of the LDL receptor in swine (22); destruction of the bacterial blight disease susceptibility gene in rice (23); replacement of a tyrosine hydroxylase gene via TALEN-enhanced homologous recombination in zebrafish (24,25)].

**Figure 1.**
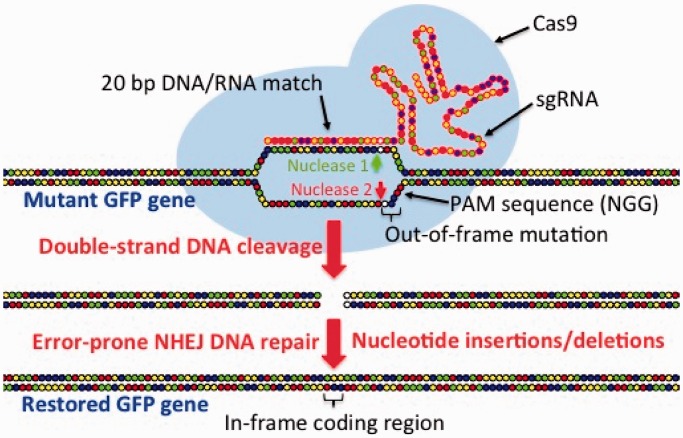
Mechanism for targeted gene disruption by the Cas9/sgRNA complex and subsequent mutagenesis by NHEJ DNA repair. Before targeted DNA cleavage, Cas9 stimulates DNA strand separation and allows a sgRNA to hybridize with a specific 20 nt sequence in the targeted gene (in this case, a non-functional, mutant GFP gene). This positions the target DNA into the active site of Cas9 in proper orientation in relation to a PAM (tandem guanosine nucleotides) binding site. This positioning allows separate nuclease domains of Cas9 to independently cleave each strand of the target DNA sequence at a point 3-nt upstream of the PAM site. The double-strand break then undergoes error-prone NHEJ DNA repair during which deletions or insertions of a few nucleotides often occurs. Those approximately one in three deletions or insertions that restore a proper reading frame in the gene’s coding region allow for restoration of gene activity.

Within the past year, another highly promising system, the clustered regulatory interspersed short palindromic repeat (CRISPR)/CRISPR-associated protein (Cas) system, has evolved from studies of a set of related defense systems that a number of bacteria use in warding off invasion by viruses and foreign DNA molecules that enter the cell (26–29). In cells containing type II CRISPR/Cas systems that successfully ward off invading viruses or foreign DNAs, 20-bp snippets of the invading DNA sometimes can be incorporated as short palindromic repeats into the CRISPR region of the bacterial chromosome. During future assaults, destruction of invading DNA is achieved by an RNA-directed process in which RNAs encoded by the short palindromic repeats of the CRISPR region are transcribed into CRISPR RNA (crRNA) segments that are processed into short, functional RNA fragments with the aid of trans-activating RNA (tracrRNA). Once processing is complete, both crRNA and tracrRNAs become associated with a Cas protein. The Cas protein contains two nuclease domains that can cleave each strand of DNA when DNA becomes associated with the Cas/crRNA/tracrRNA complex. This complex is guided to viral DNA or exogenous foreign DNA by hybridization of a 20-bp portion of the crRNA to one strand of the invading DNA. Action of the two independent nuclease domains within Cas results in a double-stranded DNA break (DSB) at a site on the foreign DNA just upstream of a protospacer adjacent motif (PAM) composed of a few (2–5) nucleotides depending on the CRISPR/Cas system involved (26). The type II CRISPR/Cas system most widely used for gene editing is derived from *Streptococcus pyogenes* (27–29) and has the advantage of possessing a PAM recognition sequence of only two nucleotides in length (GG). This allows for creation of numerous ‘guide’ RNAs (i.e. crRNAs) for any particular gene because of the usually abundant presence of GG sequences in most genes. The *S. pyrogenes* system uses a particular Cas gene, *Cas9*, that is functional as a transgene in most, if not all, cells in which it has been tested. An important innovation was the development of single guide RNAs (sgRNAs) that are fusions of critical portions of tracrRNA with the ‘guide’ and PAM domains of crRNAs (27,29) (Figure 1). Thus, to obtain a functional RNA-guided gene disruption in a host cell, one needs only to transform the cell with the *Cas9* gene and a gene (generally driven by a U6 promoter) encoding a sgRNA that contains a 20-bp sequence complementary to the segment of DNA in the host cell that is the target for disruption by a DSB. Once a host cell containing the *Cas9* gene has been established, subsequent knockout of a target gene requires only transformation with a sgRNA gene—or, if targeting of multiple genes is desired, simultaneous transformation with multiple sgRNA genes (30). Both ZFN and TALEN gene disruption systems are prone to moderate to mild cytotoxicity owing to off-site DNA cleavage and, although data are still limited, present early-phase versions of the Cas9/sgRNA system may also suffer to some degree from the same problem (31). Nonetheless, the CRISPR/Cas9 system and the simpler Cas9/sgRNA system have proven successful in gene disruption, gene activation/repression and genome editing in several cell types and organisms [e.g. in bacteria (30,32), yeast (33), zebrafish (34–36), fruit fly (37) and human cells (28,29,38)]. However, to date, there are no reports of successful expression of the CRISPR/Cas system in plants or plant cells. In this report, we demonstrate that three slightly different versions of the Cas9/sgRNA system delivered by *Agrobacterium tumefaciens* or polyethylene glycol (PEG)-mediated transfection are functional in one or more of four plant types, Arabidopsis and tobacco (dicots) and rice and sorghum (monocots). Data are presented showing successful targeting of specific DNA sequences for DNA cleavage and error-prone repair by NHEJ in rice, tobacco and Arabidopsis, the conversion of mutant out-of-reading-frame fluorescent protein genes to in-frame versions of the genes that produce readily observable green fluorescence protein (GFP) in Arabidopsis and tobacco and DsRED2 fluorescence protein in sorghum. These pioneering experiments provide compelling evidence that the Cas9/sgRNA system is fully functional in two model plant systems and two major crop species and suggest this system has promise as a powerful tool for manipulation of plant genetics in the laboratory and in the field.

## MATERIALS AND METHODS

### Plant materials

Transgenic *Arabidopsis thaliana* plants were used that conditionally express a bacterial type III effector gene, AvrPto, for suppression of host immunity. The AvrPto gene is under the control of a dexamethasone (DEX)-inducible promoter (39). Seeds containing the AvrPto gene were initially provided by Dr Fumiaki Katagiri, University of Minnesota and subsequently also purchased from ABRC (CS67140, Columbus, OH 43210, USA). Seeds were geminated and plants were grown within a controlled environment chamber at 22°C with a 12-h light/12-h dark photoperiod and ∼75% relative humidity. Wild-type *Nicotiana benthamiana* plants were grown under greenhouse conditions at ∼24°C with a 16-h light/8-h dark photoperiod and 75% relative humidity.

### Construction of plant transformation binary vectors

Constructs for expression in Arabidopsis and tobacco: For construction of the sgRNA gene, the *A. thaliana* U6-26 gene promoter and terminator regions were used (accession number At3G13855). The 5′ end of the transcript contained a 20-bp target sequence (GCGCTTCAAGGTGCACATGG) complementary to the target site in the 5′ coding region of a nonfunctional GFP gene (see description later in the text) and was followed the sgRNA scaffold (GTTTTAGAGCTAGAAATAGCAAGTTAAAATAAGGCTAGTCCGTTATCAACTTGAAAAAGTGGCACCGAGTCGGTGCTTTTTTT) (29). The complete gene was synthesized by GenScript (GenScript.com). The sequence of the synthetic sgRNA gene transcript is provided in Supplementary Data.

For construction of the Cas9 gene, the Cas9 coding region from *S. pyogenes* (4014 bp, GeneID:3572134) was codon optimized for expression in *Chlamydomonas reinhardtii* and synthesized by GenScript. The coding region of Cas9 was fused in frame with a 2XFLAG (GATTACAAGGACGATGATGACAAGAAAGACTATAAAGATGACGATGATAAGCAT) at the 5′ terminus immediately downstream of the ATG start codon and with a SV40 nuclear localization sequence (CCGAAGAAGAAGCGCAAGGTGTAA) at the 3′ end of the gene (29). The chimeric Cas9 gene was driven by the CaMV35S promoter and terminated by the nopaline synthase (NOS) gene terminator region (TNOS). The sequence of the Cas9 gene construct is provided in Supplementary Data.

GFP (monomeric GFP fluorescent protein gene, Accession Number AAC53663) was used as reporter for the Cas9/sgRNA system. The coding region of GFP gene was driven by the CaMV35S promoter and terminated with TNOS. A target sequence complementary to the 20-nt sequence in the sgRNA described earlier in the text plus a PAM sequence (AGG) (GCGCTTCAAGGTGCACATGGAGG) was inserted just downstream of the ATG start codon at the 5′ terminus of the GFP gene and caused the GFP coding region to be shifted out-of-frame. An *ApaLI* restriction enzyme recognition site (underlined in the sequence earlier in the text) was included in the 20-bp target site just downstream of the PAM site (i.e. 2 nt away from the presumed Cas9 cut site within the target DNA sequence) to allow enrichment of mutagenized DNA sequence in a scheme described later in the text. The DNA sequence of the GFP reporter gene is provided in Supplementary Data.

For construction of the plant transformation binary vectors containing Cas9, sgRNA and snonfunctional GFP genes, a pCAMBIA 1302 vector (for details, see pCAMBIA Vectors, http://www.cambia.org/daisy/cambia/585) was used. The original MCS site of pCAMBIA 1302 was destroyed by *EcoRI-HindIII* digestion and self-ligation. The region between *SpeI* site and 37-bp upstream of the RB was PCR modified to contain new restriction enzyme cut sites (i.e. 5′ *SpeI-HindIII-SacI-EcoRI-SalI-PstI-KpnI* 3′). The Cas9 gene expression cassette was inserted in a 5′–3′ orientation into this modified pCAMBIA 1302 vector using *SpeI-SacI* sites. The sgRNA gene was inserted (also with a 5′–3′ orientation) into the *SalI-KpnI* sites. The non-functional GFP gene cassette was inserted into a separate pCAMBIA 1302 vector at the *NcoI-BstEII* sites. These two binary vectors (see T-DNA region sequence and vector map in Supplymentary Information) were each transformed into a separate *A. tumefaciens* host strain, one into C58 and the other into EHA105.

### Enrichment of mutagenized GFP genes

To verify Cas9/sgRNA-stimulated cleavage by the Cas9/sgRNA complex in transformed Arabidopsis and tobacco leaf cells and subsequent mutagenic NHEJ DNA repair, DNA was extracted from *Agrobacterium*-inoculated leaf areas, 48 h after infiltration, for PCR amplification. A NucleoSpin Plant II kit (MACHEREY-NAGEL GmbH and Co.KG, Germany) was used for DNA extraction. To eliminate PCR amplification of most DNA sequences from the original non-functional GFP gene, 1 µg of the extracted DNA was cleaved at the resident *ApaLI* cut site that is located only 2 bp away from the presumed Cas9/sgRNA cleavage site. In this way, subsequent PCR amplification favored production of DNA products from GFP genes that had been mutagenized by combined Cas9/sgRNA cleavage and error-prone NHEJ DNA repair in a manner that destroyed the *ApaLI* restriction site. For PCR amplification, primers 125-bp upstream and 125-bp downstream of the *ApaLI* restriction site were used to amplify ∼40 ng of*ApaLI*-digested DNA. Sequences of the primers used are provided in Supplementary Information. The PCR reaction was conducted using Phusion High-Fidelity DNA Polymerase (Finneymes, product code F-530 S) with annealing conditions of 60°C for 30 s, reaction conditions of 72°C for 30 s and denaturing conditions of 98°C for 10 s over 36 cycles. PCR amplified DNA was agarose gel-purified and subjected to an additional round of *ApaLI* digestion before cloning into a BlueScript vector. DNA sequencing of the cloned DNA was used to determine the types of Cas9/sgRNA/NHEJ-mediated mutations obtained.

### Arabidopsis and tobacco leaf cell transformation by *Agrobacterium* infiltration

*Agrobacterium* strain C58 (carrying the Cas9 and sgRNA genes) and strain EHA105 (carrying the nonfunctional GFP gene) were grown overnight at 27°C with shaking at 200 RPM in 5 ml of Luria–Bertani medium supplemented with appropriate antibiotics. The next day, 1.5 ml of bacterial culture at saturation density was harvested by centrifugation and washed once with 1.5 ml 1× Infiltration Buffer [42.6 g of (2-(N-morpholino)ethanesulfonic acid (MES), 20 g of d-Glucose and 3.04 g of Na_3_PO_4_·12H_2_O in 400 ml of water (pH 5.6)] (39). The bacterial pellet was washed again by suspension in 1× Infiltration Buffer with 0.1 µM acetosyringone and diluted with the same buffer to OD_600_ = 0.2. The two individual bacteria were mixed 1:1 immediately before leaf infiltration. For Arabidopsis leaf cell transformation, 4-week-old seedlings of transgenic plants containing a DEX-inducible AvrPto gene (39) were sprayed with 10 µM DEX in 0.01% Silwet L-77. Plants were maintained at 23°C for 24 h before inoculation. Arabidopsis leaves ∼1–1.5 cm in width were infiltrated with a mixture of two *Agrobacterium* strains in a volume of 20–50 µl per leaf using a 1 ml of syringe without a needle.

### Fluorescence confocal microscopy of Arabidopsis and tobacco leaf cells

Two to three days after infiltration, leaves were cut and analyzed for GFP signal using a Nikon ECLIPSE 90i system confocal fluorescence microscope at 40× and 600× magnification. The excitation and detection wavelengths were set at 448 nm and 500–550 nm, respectively, for GFP fluorescence and at 641 nm and 662–737 nm for chlorophyll auto-fluorescence to ensure a lack of cross-talk between the two fluorescence channels. Similar to Arabidopsis, leaves of 4-week-old *N. benthamiana* plants were infiltrated with a mixture of the two *Agrobacterium* strains and subject 2 days later to fluorescence microscopy analysis.

### Immature sorghum embryo transformation with Cas9 and sgRNA genes

Y158, a monocot expression vector for testing Cas9 and sgRNA gene activity in sorghum immature embryos, contained the following expression cassettes: The maize ubiquitin 1 promoter/intron combination (40) expresses a red fluorescence protein (DsRED2) coding region with a nopaline synthase 3′ end. The DsRED2 is intentionally designed to be out of frame and contains a target for a Cas9 guide RNA. Downstream of the DsRED2 chimeric gene is a rice Actin 1 promoter/intron combination (41) expressing a synthetic Cas9 coding region. The synthetic Cas9 coding region contains monocot preferred codons for higher expression in monocots. The octopine synthase 3′ polyadenylation region is downstream of the Cas9 coding region. A 516-bp rice U6 promoter identical to the U6 promoter (Genbank AL731888.5) was PCR amplified from Nipponbare genomic DNA and attached to a sgRNA gene with a 20-nt sequence targeting the DsRED2 gene at its 5′ end (Supplementary Data). These three gene cassettes (DsRED2 target, Cas9 and sgRNA) are contained within the T-DNA region of a pVS1 binary vector derived from pLH7500 (GenBank: AY234331.1). This binary vector also contains a GFP(cloverFP)-NptII fusion gene expressed from the CaMV 35 S promoter and maize hsp70 intron as a visual/selectable marker gene.

Two weeks after *Agrobacterium*-mediated transfer of the T-DNA of Y158 to immature sorghum embryos (42), stably transformed groups of cells expressing GFP are observable.

### Constructs for Cas9 and sgRNA gene expression in rice

The *S. pyogenes* Cas9 (SpCas9) coding sequence from plasmid pMJ806 (Jinek *et al.*, 2012, obtained from Addgene, http://www.addgene.org/) was PCR amplified with primers Cas9-F1 and Cas9-R1 (Supplementary Information) that contain restriction sites *BglII* and *SpeI*, respectively. The amplicon was cloned into pGEM-T (Promega) A/T cloning vector and sequenced for accuracy. The Cas9 gene was then cloned into an intermediate vector that contained sequences encoding a nuclear localization signal and 3XFLAG sequence (MAPKKKRKVGDYKDHDGDYKDHDIDYKDDDDK) at the 3′-terminus. The chimeric Cas9 was further cloned downstream of the CaMV 35 S promoter and upstream of a NOS gene termination region in a vector containing a pUC19 backbone sequence. Similarly, synthetic Cas9 (produced by GenScript) with optimized rice codons (OsCas9) was cloned in the same expression vector.

The sgRNA was constructed in a manner similar to that for the GFP sgRNA described earlier in the text except that the seed sequence (5′- GAGCTTAGCACCTGGTTGGAGGG -3′) targeting the promoter of *OsSWEET14* and a shorter sgRNA scaffold following the seed sequence (GTTTTAGAGCTAGAAATAGCAAGTTAAAATAAGGCTAGTCCGTTTTT) were used. The promoter and terminator of a rice U6 gene along with a cloning sequence for sgRNA were constructed by using an overlap PCR method with primers U6P-F1 & U6P-R1 and U6P-F2 & U6P-R2 (Supplementary Information). The PCR product was cloned into pTOPO/D (Invitrogen), resulting in pTOPgRNA. The chimeric sgRNA for *OsSWEET14* was constructed by cloning annealed oligos crRNA4-F (5′-GTTGAGCTTAGCACCTGGTTGGA-3′) and crRNA4-R (5′-AAACTCCAACCAGGTGCTAAGCT-3′) into pTOPgRNA predigested with *BsaI*. Similarly, the sgRNA for *OsSWEET11* was constructed into pTOPgRNA with the oligos crRNA5-F (5′ – gttGACTTTTGGTGGTGTACAGTA -3′) and crRNA5-R (5′- AAACTACTGTACACCACCAAAAGT -3′).

### Transient expression of Cas9/gRNA in rice protoplasts

The protocol for rice mesophyll protoplast isolation and transfection described by Zhang *et al.* (43) was adopted with minor modifications. Briefly, surface-sterilized rice seeds were germinated on solid ½ MS medium at 28°C under a 12-h light/12-h dark cycle. The stems and sheathes of seedlings (∼7 days old) were cut into small strips (∼0.5 mm) and incubated in solution containing cell wall digesting enzymes [1.5% cellulose RS, 0.75% macerozyme R-10, 0.6 M mannitol, 10 mM MES, 1 mM CaCl2, 5 mM β-mercaptoethanol and 0.1% BSA (pH 5.7)] for 6–8 h in the dark with gentle shaking. The released protoplasts were collected and washed with W5 solution [154 mM NaCl, 125 mM CaCl2, 5 mM KCl and 2 mM MES (pH 5.7)] before transfection. A mixture of Cas9 gene and sgRNA gene constructs (10 µg with a 1:1 molar ratio) was used to transfect 200 µl (4 x 10^5^ cells) of protoplasts using the PEG-mediated DNA delivery method described by Zhang *et al.* (2011). Transfected protoplasts were incubated in dark at 28°C for 48 h before collection for genomic DNA extraction using a hexadecyltrimethylammonium bromide (CTAB) method (44).

### Mutation detection in DNA from rice protoplasts

For *OsSWEET14*, genomic DNA extracted from protoplasts transfected with SpCas9/sgRNA gene constructs was treated with the restriction enzyme, *SexAI*, to allow for enrichment of mutated alleles that had lost the *SexAI* restriction enzyme site due to SpCas9/sgRNA-mediated mutagenesis at the *OsSWEET14* gene target site. The *SexAI* treated DNA was used for PCR amplification of the *OsSWEET14* promoter region with primers SWT14-F (5′- CACCGGTACCATGGCTGTGATTGATCAGG-3′) and SWT14-R (5′- TGCAGCAAGATCTTGATTAACTAG-3′). For *OsSWEET11*, genomic DNA from OsCas9/sgRNA treated cells was predigested with *BsrGI* before PCR-amplication with primers SWT11-F (5′ -TGAGTGGTCATACGTGTCATATTG -3′) and SWT11-R (5′ - CCGGATCCATTGCTACTGGTGATGAAGGT -3′). The resulting amplicons of *OsSWEET14* and *OsSWEET11*, respectively, were cloned into pGEM-T (Promega). Clones containing sequences of approximately the expected size were sequenced to detect potential mutations in the two Cas9/sgRNA-targeted SWEET gene promoter regions.

## RESULTS AND DISCUSSION

### Strategy for detection of Cas9 and sgRNA activity in Arabidopsis and tobacco

**Figure 2.**
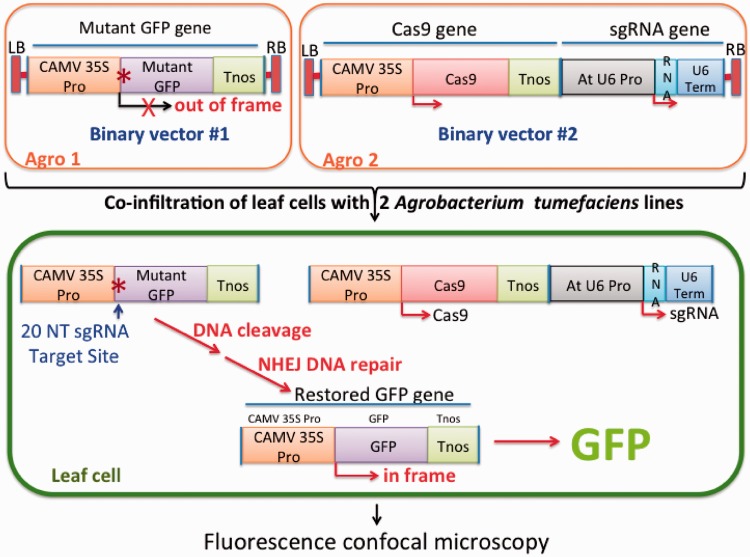
Strategy for use of the Cas9/sgRNA system for mutagenesis and restoration of activity of a non-fuctional mutant GFP gene following Agrobacterium-mediated delivery of the Cas9 and sgRNA genes to Arabidopsis or tobacco cells. The Cas9 and sgRNA genes reside on a single binary vector that is carried by one *A. tumefaciens* line and the mutant GFP gene is present on a different binary vector in a separate *A. tumefaciens* line. Following infiltration of leaves with a mixture of the two *A. tumefaciens* lines and co-transformation of single cells, a specific 20-nt DNA sequence in the mutant GFP gene is targeted by the Cas9/sgRNA complex for cleavage. Repair of the double-strand DNA break by NHEJ results in deletion or insertion of nucleotides at the cleavage site and, in some case, restoration of a functional GFP gene, the product of which can be observed by fluorescence confocal microscopy.

Cleavage of double-stranded DNA caused by Cas9/sgRNA expression most likely will be repaired by the NHEJ DNA repair mechanism, an error-prone process in which repair is often accompanied by small deletions or insertions of nucleotides at the site of repair. We took advantage of this situation to create a reporter system for detecting Cas9/sgRNA activity in Arabidopsis and tobacco leaves simultaneously expressing the Cas9 and sgRNA genes. The reporter system (outlined in Figure 2) involved a non-functional mutant version of a GFP gene containing a small 20 nt insertion immediately downstream of the ATG start codon that caused a shift in the reading frame of the gene and, because of a nearby downstream stop codon, the production of a short truncated polypeptide lacking the ability to produce a fluorescent signal in transgenic plant cells. Successful binding of the 20 nt sequence by Cas9 guided by a sgRNA complementary to the 20 nt target and cleavage of the DNA in some cases would stimulate NHEJ DNA repair and the insertion or deletion of a stretch of nucleotides that would reestablish a correct reading of the GFP gene and the production of a green fluorescent signal in transgenic plant cells. To eliminate the unlikely possibility that inadvertent expression of the Cas9 and sgRNA genes in a single bacterial cell might allow for Cas9/sgRNA cleavage of the target DNA sequence and restoration of a functional GFP gene within the bacterial cell, we designed our system to place the mutant GFP gene on one binary vector and the sgRNA gene along with the Cas9 gene on a different binary vector and introduce these two binary vectors into two separate *A. tumefaciens* lines. Following leaf infiltration with these two lines, only cells genetically transformed with both lines would contain all three genes (mutant GFP, Cas9 and sgRNA genes) needed to create a functional GFP reporter gene (Figure 2).

#### Cas9/sgRNA activity in Arabidopsis leaf cells

**Figure 3.**
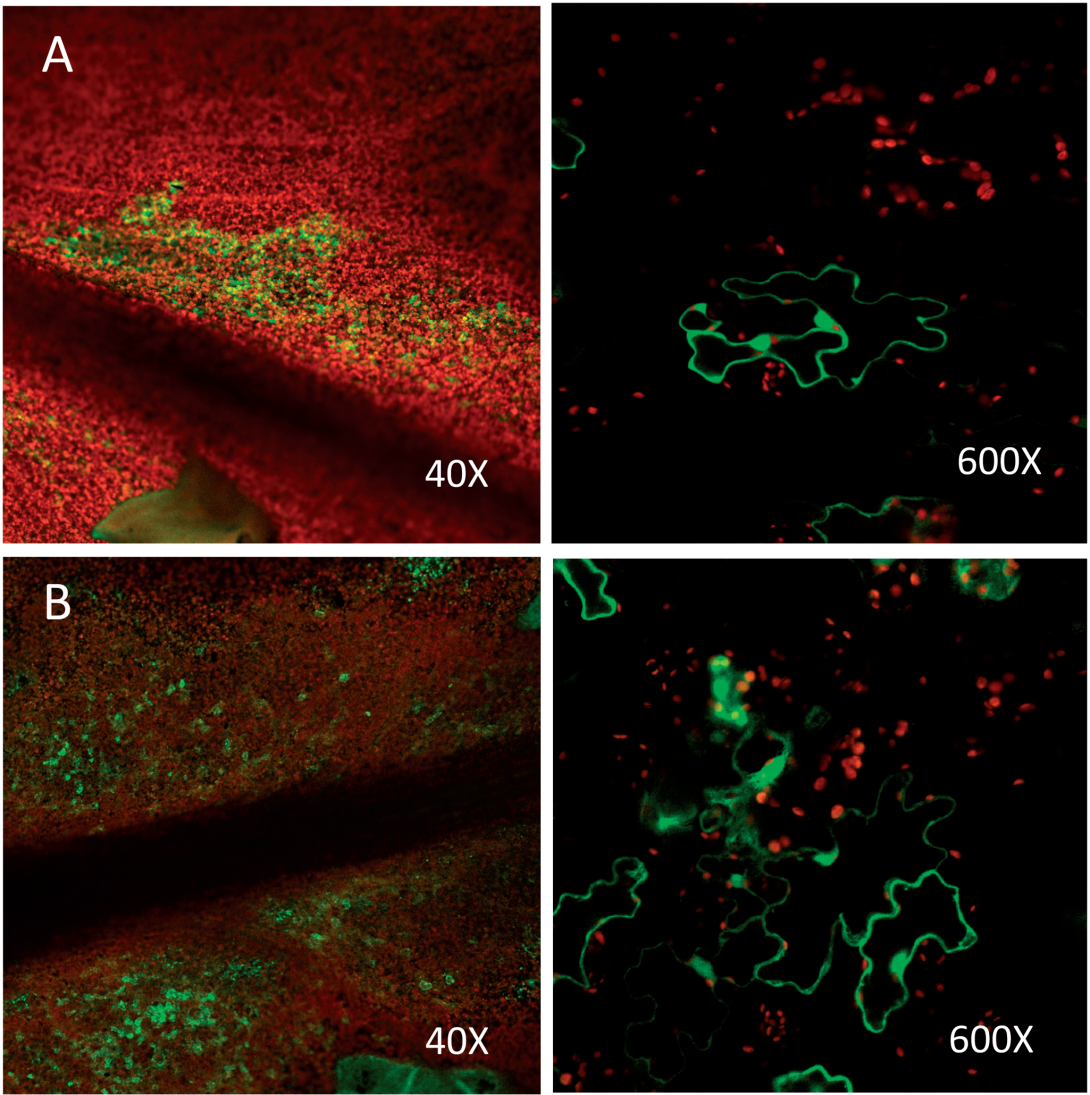
Red chlorophyll fluorescence signals and GFP signals from Arabidopsis leaf cells. (**A**) Leaf cells infiltrated with *A. tumefaciens* carrying a vector containing a wild-type GFP gene. (**B**) Leaf cells infiltrated with a mixture of two *A. tumefaciens* lines, one with a vector containing a non-functional mutant GFP gene and the other line carrying a vector for the expression of the Cas9 gene and the sgRNA gene. Expression of GFP in these leaf cells can occur only if a DNA target site in the non-functional GFP is recognized and cleaved by a Cas9/sgRNA complex, and resulting error-prone DNA repair by NHEJ leads to deletion or insertion of nucleotides that restore a proper reading frame in the GFP gene-coding region. Leaves were photographed at 40× and 600× magnification 48 h after *A. tumefaciens* infiltration.

To obtain efficient transformation of leaf cells with *A. tumefaciens*, we used *A. thaliana* plants that conditionally express, when treated with DEX, the bacterial AvrPto effector that suppresses host immunity (39). In control experiments, *A. tumefaciens* containing a binary vector carrying a functional GFP gene driven by the CaMV 35 S promoter was used to infiltrate Arabidopsis leaves and resulted in the production of patches of cells displaying strong green fluorescence when examined by confocal fluorescence microscopy (Figure 3A). To determine whether Cas9/sgRNA activity could be obtained in Arabidopsis, leaves were simultaneously infiltrated with two *A. tumefaciens* lines, one containing a binary vector carrying both the Cas9 gene and a sgRNA gene driven by an Arabidopsis U6 gene promoter and another *A. tumefaciens* line containing a binary vector carrying the out-of-frame GFP gene. The sgRNA gene was designed to produce a sgRNA capable of binding a 20-nt target sequence in the 5′ region of the mutant GFP gene (Figure 2). In ∼20% of the leaves examined 48 h after infiltration, several patches of cells could be observed with confocal fluorescence microscopy to display strong green fluorescence (Figure 3B). In many cases, the patches of fluorescence were similar in number and intensity to those patches observed when leaves were inoculated with *A. tumefaciens* carrying a wild-type GFP gene (Figure 3A). The intensity of fluorescent signals emanating from leaves inoculated with *A. tumefaciens* containing either wild-type GFP gene or mutant GFP gene constructs was maintained at moderate-to-high levels for at least 10 days following the initial infiltration.

Arabidopsis leaves infiltrated only with *A. tumefaciens* containing the mutant GFP gene all failed to produce fluorescent signals. More critically, no green fluorescent signals were obtained when leaves on 30 different Arabidopsis plants were infiltrated with the same two strains of *A. tumefaciens* used in the experiments depicted in Figure 3B with the exception that the U6 promoter was used to drive a sgRNA gene containing a random 20-nt ‘guide’ sequence that did not match any GFP gene sequences or any Arabidopsis genome sequence. Together, these experiments demonstrate that the *Agrobacterium*-mediated gene delivery procedure, per se, did not cause frame-shift mutations in the mutant GFP gene and, more to the point, that Cas9 and target-specific sgRNA genes are essential for obtaining mutagenesis of the non-functional GFP gene.

### Cas9/sgRNA activity in tobacco leaf cells

**Figure 4.**
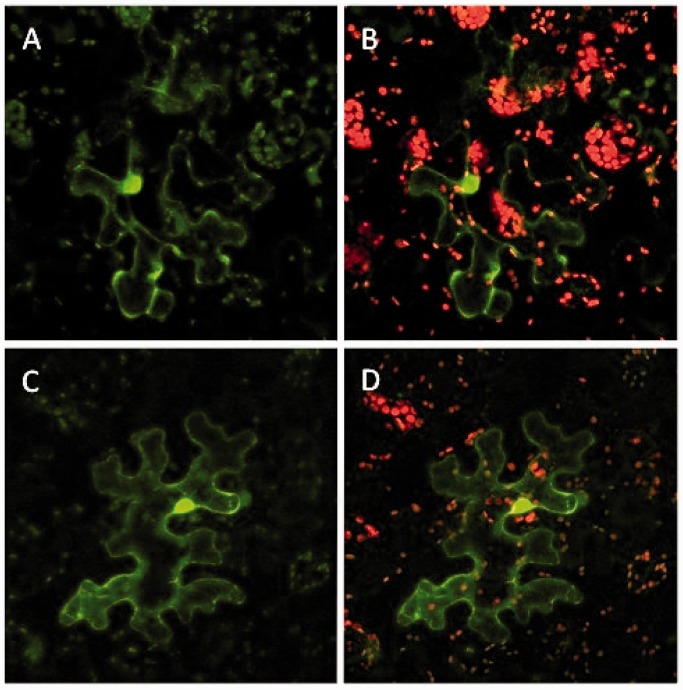
Red chlorophyll fluorescence signals and GFP signals from tobacco leaf cells. (**A** and **C**) Two examples of tobacco leaf cells infiltrated with a mixture of two *A. tumefaciens* lines, one with a vector containing a nonfunctional, mutant GFP gene and the other line carrying a vector for the expression of the Cas9 gene and the sgRNA gene. Green fluorescence is due to restoration of a proper reading frame of the GFP gene due to Cas9/sgRNA-mediated targeted gene cleavage and subsequent error-prone NHEJ DNA repair. (**B** and **D**) The images displayed in (A) and (C) merged with images of red fluorescence from chlorophyll. Leaves were photographed at 600× magnification 48 h after *A. tumefaciens* infiltration.

In experiments identical in design to those carried out with Arabidopsis leaves, we infiltrated *N. benthamiana* leaves with the same combination of *A. tumefaciens* lines described earlier in the text. Figure 4 displays two separate transformation results in tobacco leaves in which co-delivery of Cas9, sgRNA and non-functional GFP genes by *A. tumefaciens* resulted in modification of the GFP gene and restoration of GFP gene function. The images of Arabidopsis and tobacco leaf cells depicted in Figures 3B and 4, respectively, show prominent localization of green fluorescence in cell nuclei. This pattern of fluorescence is distinct from autofluorescence produced by dying cells or cells infected with pathogens in which autofluorescence is generally confined to the cytoplasm.

### DNA sequence analyses of Cas9/sgRNA-induced mutations in the non-functional GFP gene occurring in Arabidopsis and tobacco leaf cells

**Figure 5.**
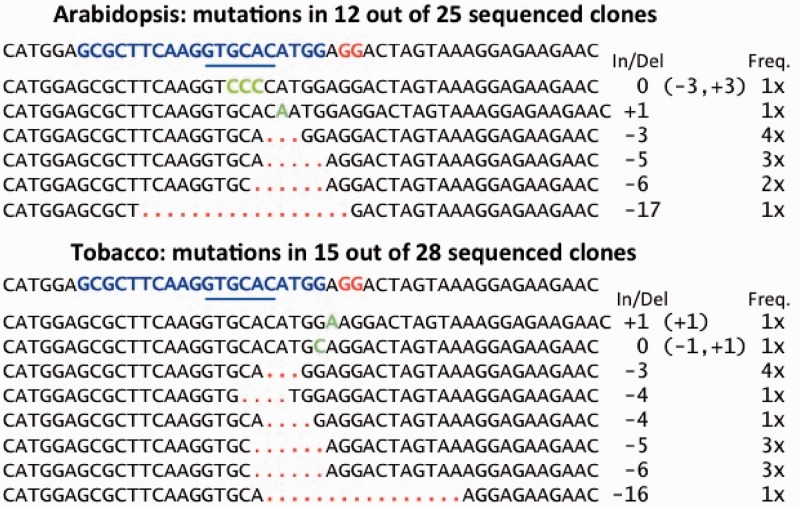
DNA sequences of initially non-functional GFP genes following target site cleavage by Cas9/sgRNA and mutagenesis by NHEJ DNA repair in transgenic Arabidopsis and tobacco leaf cells expressing Cas9 and sgRNA genes. The DNA sequence of the starting non-functional mutant GFP gene is provided above each set of Cas9/sgRNA-mutagenized gene sequences derived from Arabidopsis (top set) and tobacco (bottom set). The 20 nt target sequence for the Cas9/sgRNA complex is in blue, the PAM site in red, and the *ApaLI* recognition site is underlined in blue. For the Cas9/sgRNA-mutagenized DNA sequences, deleted nucleotides are depicted as red dots and inserted nucleotides are shown in green. The net length of insertions and/or deleletions (In/Del) and the frequency with which each DNA sequence pattern was observed (Freq.) are presented in the columns to the right. One sequence in the Arabidopsis set and one sequence in the tobacco set arose from sequential deletions and insertions (first and second lines of each DNA sequence set, respectively).

To establish that the Cas9/sgRNA system causes mutations at the target site in the non-functional GFP genes in Arabidopsis and tobacco leaf cells is responsible for the conversion of the non-functional GFP gene into a functional GFP gene, DNA was extracted from leaf regions displaying fluorescence and subjected to PCR amplification using primers annealing to sites 125-bp upstream and 125-bp downstream of the Cas9-sgRNA target site. By digesting the extracted DNA with the restriction enzyme, *ApaLI* before amplification, advantage was taken of the presence of a *ApaLI* restriction site at the targeted Cas9-sgRNA cut site of the nonfunctional GFP to prevent the amplification of DNA from the non-functional GFP gene but allow amplification of mutagenized GFP gene segments in which the *ApaLI* cut site had been destroyed by nucleotide insertions or deletions created during DNA repair by the NHEJ repair system. DNA sequencing of cloned PCR products showed that approximately one-half of the cloned DNA sequences were from non-mutagenized non-functional GFP genes that apparently escaped *ApaLI* digestion (Figure 5). Nonetheless, the procedure for enrichment of mutagenized GFP gene DNA segments was successful in allowing recovery of clones of PCR-amplified DNA bearing six different DNA sequence patterns from Arabidopsis leaves and eight different DNA sequence patterns from tobacco leaves (Figure 5). As is common with NHEJ DNA repair, most mutations resulted from small deletions ranging from 1 to 17 nt or insertion of 1 to 3 nt. In two cases, there were apparent combinations of small deletions and insertions.

Because it is reported that Cas9 nucleases cleave 3 nt upstream of the PAM sequence (27), it is likely that our *ApaLI*-mediated enrichment for GFP target site mutations may well have resulted in lack of recovery of a number of target region mutations. This is because, in our non-functional GFP gene, Cas9-sgRNA cuts the target DNA two nucleotides downstream of the *ApaLI* recognition sequence. Accordingly, after Cas9-sgRNA-mediated cleavage and subsequent mutagenic NHEJ DNA repair, there likely are deletions or insertions that do not reach into the *ApaLI* site—and, thus, the resulting mutagenized DNA sequences are eliminated by *ApaLI* digestion from PCR amplification and DNA fragment cloning. Regardless, it is clear that the Cas9-sgRNA system is fully functional in DNA cleavage and triggering mutagenic gene disruption in both Arabidopsis and tobacco leaf cells.

### Cas9/sgRNA activity in sorghum immature embryos

**Figure 6.**
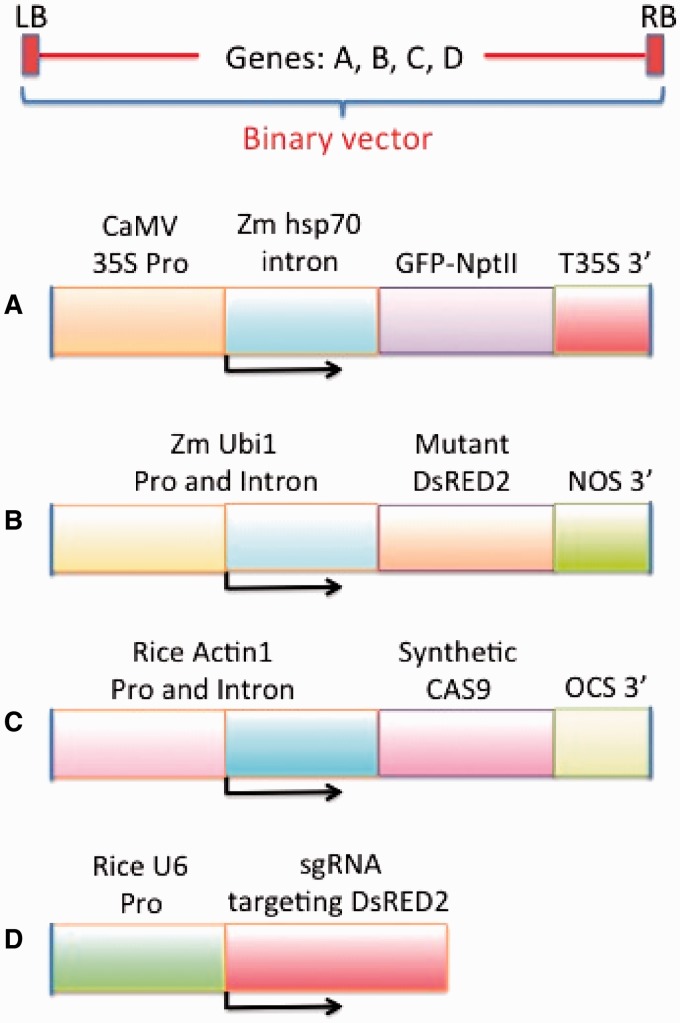
Gene constructs in a binary vector used in transformation of immature sorghum embryos to test for Cas9/sgRNA activity. A single binary vector was designed and constructed to contain (**A**) a chimera of a wild-type GFP gene and a neomycin phosotransferase gene, (**B**) a non-functional mutant DsRED2 gene, (**C**) a Cas9 gene codon-optimized for expression in maize, (**D**) a sgRNA gene driven by a rice U6 promoter and targeting the mutant DsRED2 gene.

In a strategy similar to that used above with Arabidopsis and tobacco, an *A. tumefaciens* binary vector, Y158, was designed and constructed that carries four independent genes—an out-of-frame red fluorecence protein gene (DsRED2) (the target for Cas9-sgRNA cleavage and mutagenesis), a synthetic Cas9 gene codon optimized for expression in monocots, a U6 promoter-driven sgRNA gene and a GFP-NptII fusion visual/selectable marker gene (Figure 6). The maize ubiquitin 1 promoter/intron combination expresses a DsRED2 coding region with a nopaline synthase (NOS) 3′ end. The DsRED2 is intentionally designed to be out of frame and contains a target for the Cas9/sgRNA complex near the beginning of the DsRED2 coding region. Downstream of the DsRED2 chimeric gene is a rice Actin 1 promoter/intron combination expressing a synthetic Cas9 coding region. The octopine synthase 3′ polyadenylation region is downstream of the Cas9 coding region. A rice U6 promoter is used to express a U6 transcript as a guide RNA with a targeting sequence at its 5′ end. These three gene cassettes (DsRED2 target, Cas9 and sgRNA) are contained within the T-DNA region of a pVS1-derived binary vector. The T-DNA region of this binary vector also contains a GFP-NptII fusion gene expressed from the CaMV 35 S promoter and maize hsp70 intron. The expression of the GFP-NptII fusion protein allows transformed cells to be identified by their GFP expression.

**Figure 7.**
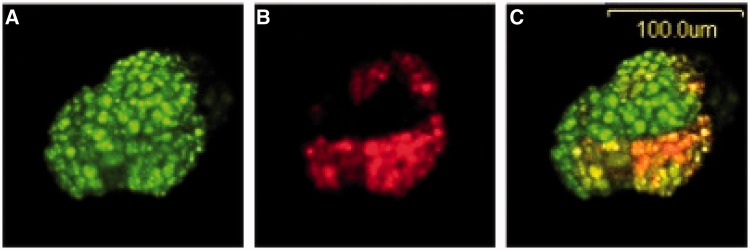
Clover (green) fluorescence protein signals and DsRED2 fluorescence protein signals from immature sorghum embryo cells. An immature sorghum embryo was co-cultivated with *A. tumefaciens* cells carrying a binary vector containing four genes driven by plant gene promoters (i.e. a clover fluorescence gene, a mutant non-functional DsRED2 gene containing a 20-bp CAs9/sgRNA target site, a Cas9 gene and a sgRNA gene driven by a rice U6 gene promoter) as described in Figure 6. (**A**) Image of cells of the immature embryo expressing the clover fluorescence protein. (**B**) Image of cells expressing red fluorescence protein produced from a mutagenized nonfunctional DsRED2 gene converted by the action of a Cas9/sgRNA complex and subsequent NHEJ into a functional DsRED2 gene. (**C**) Merged image of A and B confirming expression of clover fluorescence protein in a cluster of adjacent immature embryo cells and co-expression of clover fluorescence protein and DsRED2 fluorescent protein in an immediately adjacent cluster of transformed cells. Embryo cells were photographed 2 weeks following co-cultivation with *A. tumefaciens* carrying the Y158 binary vector.

Two weeks after *Agrobacterium*-mediated transfer of the T-DNA of Y158 to immature sorghum embryos, stably transformed groups of cells expressing GFP are observable. Approximately 1/3 (5 of 18) of the stably transformed GFP positive groups of cells contained DsRED2 sectors (Figure 7). These DsRED2 sectors were a subset of the GFP positive cells in all five instances. This frequency of NHEJ in the stably transformed groups of immature embryo cells approaches the one-third maximum frequency possible due to the 1-in-3 chance of restoring the DsRED2 reading frame during NHEJ, indicating a high frequency of Cas9/sgRNA targeting. This sectoring of the DsRED2 within a subset of the multiple GFP positive cells suggests the NHEJ reaction occurred, in this particular case, only after one or more divisions of cells expressing the GFP gene. This sectoring therefore suggests that Cas9/sgRNA-directed gene cleavage and NHEJ occurred in a gene that was stably integrated into the plant cell’s chromosome.

In principle, a bacterial Cas9-induced NHEJ could occur if both the Cas9 and U6 guide RNA are expressed in bacteria. If a hypothetical NHEJ occurred, it should result in GFP and DsRED2 expression in all the stably transformed cells derived from the stable integration of this T-DNA. None of the 18 GFP-positive groups of cells were observed to have uniform DsRED2 expression, indicating NHEJ is likely not occurring in bacteria containing the Y158 binary vector. The complete separation of the Cas9 gene and the sgRNAgene in one *A. tumefaciens* line and the non-functional GFP gene in a different *A. tumefaciens* line (Figure 2) during the Arabidopsis and tobacco transformations described earlier in the text preclude the possibility that Cas9-sgRNA-mediated GFP gene mutagenesis is occurring in a single bacterial cell and, thus, provides compelling evidence that Cas9-sgRNA activity resides exclusively in the plant cell. Likewise, the delivery of Cas9 and sgRNAs by PEG-mediated uptake into rice protoplast cells described later in the text and subsequent Cas9/sgRNA-mediated target gene mutagenesis involve no bacterial participation and, therefore, dictates that the Cas9/sgRNA complex is active within the plant cell.

### Cas9/sgRNA activity in rice protoplasts

**Figure 8.**
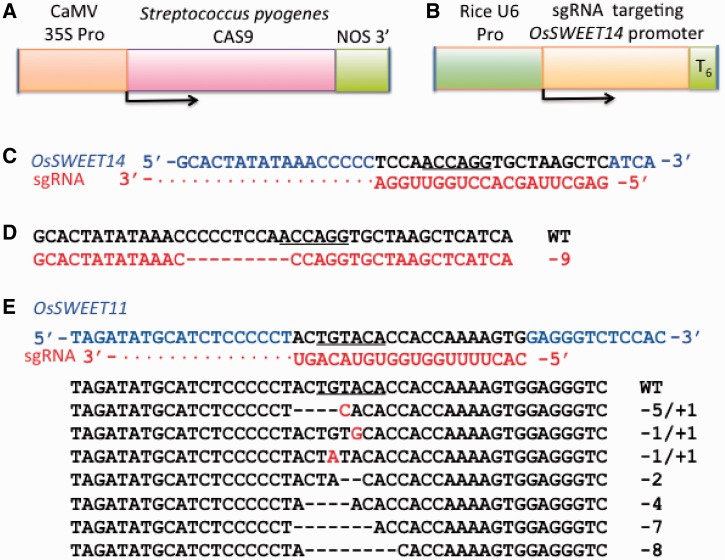
Gene constructs used in transformation of rice protoplasts to test for Cas9/sgRNA activity and test results. (**A**) The *S.pyogenes* Cas9 gene driven by the CaMV 35S promoter and containing a Nos gene termination region. (**B**) A synthetic single guideRNA gene targeting the promoter region of the rice *OsSWEET14* gene and containing a hexathymidine U6 gene termination signal. (**C**) Diagram showing the 20-nt sequence of the *OS11N3* gene (black) that is the target for hybridization to 20 nt in the synthetic sgRNA molecule (red). The *SexAI* restriction enzyme recognition site is underlined. (**D**) Diagram showing the DNA sequence of the target region of a *OsSWEET14* gene (red) in which Cas9/sgRNA-mediated DNA cleavage and subsequent error-prone NHEJ DNA repair have caused mutagenesis leading to deletion of 9 nt (red dashes) from a critical region of the *OsSWEET14* gene promoter. *SexAI* DNA recognition sequence is underlined in the wild-type sequence. (**E**) Diagram showing the DNA sequence of the target region of*OsSWEET11* gene in which Cas9/sgRNA-mediated DNA cleavage and subsequent error-prone NHEJ DNA repair have caused mutagenesis leading to deletion (dashes) and substitutions (red letters) at the expected sgRNA target site in the *OsSWEET11* gene promoter. The DNA sequence recognized by *BsrGI* is underlined in the wild-type sequence.

Because endonucleases such as TALENs have been shown to be effective in causing site-specific DSBs and subsequent genetic mutations in rice (23,45), we also tested whether the Cas9/sgRNA system was able to induce DNA alterations in rice cells. For this purpose, two constructs were produced, one encoding the *S. pyogenes* Cas9 enzyme (non-codon optimized for rice) under control of the CaMV 35 S promoter and another expressing a chimeric sgRNA, driven by a rice U6 gene promoter, containing a 5′ region complementary to a sequence of 20 bp in the promoter of *OsSWEET14* and a 3′ sgRNA scaffold for recruiting Cas9 (Figure 8A–C). Rice protoplasts were co-transformed with a mixture of the two constructs using a PEG-based transformation protocol and were incubated for 48 h to allow Cas9/sgRNA alteration of the targeted endogenous *OsSWEET14* locus. Genomic DNA was extracted from pooled cells and treated with *SexAI*, which recognizes and cleaves ACCAGG (a site 1 base pair downstream of the expected Cas9 cleavage site) to allow enrichment for mutated alleles lacking an intact *SexAI*-recognition sequence. The *SexAI*-resistant genomic DNA was used for PCR-amplification of the *OsSWEET14* promoter region, and the amplicons were cloned into an A/T cloning vector for DNA sequencing. Sequencing of eight clones revealed seven amplicons that contained a 9-bp deletion and one that contained a wild-type promoter sequence (Figure 8D). The deletion is located in close proximity to the predicted cleavage site of the Cas9/sgRNA complex [3-bp downstream of PAM sequence (27)], indicating the association of the mutation with Cas9/sgRNA-directed DNA cleavage.

To determine whether codon optimization of the Cas9 gene would influence rates of Cas9/sgRNA-directed mutagenesis, an experiment was conducted targeting a separate rice endogenous gene, *OsSWEET11,* for mutagenesis using a Cas9 gene optimized for expression in rice (OsCas9, Supplementary Information). In this experiment, several target site mutations were obtained (Figure 8E). Again, a mutation enrichment approach was used that was similar to that used with the *OsSWEET14* except that the genomic DNA from pooled protoplasts transfected with 35 S-OsCas9 and sgRNA was digested with *BsrGI* (present in the target site of *OsSWEET11*; Figure 8E) and used for PCR-amplification of the sgRNA target region. The amplicons were cloned into an A/T cloning vector for DNA sequencing. Sequencing of 11 clones revealed 10 amplicons that contained seven distinct mutations and one that contained a wild-type promoter sequence (Figure 8E). It may be noted, as with the similar enrichment strategy described earlier in the text for Arabidopsis and tobacco DNA, that the *SexAI* and *BsrGI* digestion and enrichment strategies used in rice are capable of recovery of only a portion of mutations generated by the Cas9/sgRNA system.

## CONCLUSIONS

Data presented in this report provide unambiguous evidence that the Cas9/sgRNA system for targeted gene modification functions in higher plant cells. Expression of Cas9 and sgRNA genes in two dicot species, Arabidopsis and tobacco, led to targeted cleavage of a non-functional GFP gene, mutagenesis by NHEJ DNA repair (verified by DNA sequencing) with restoration of a correct reading frame to the GFP gene and production of a strong green fluorescence in transformed leaf cells. Rates of transformation approached that obtained with infiltration of *A. tumefaciens* carrying a binary vector containing a wild-type GFP gene. Similar mutagenic effects of the Cas9/sgRNA system in immature sorghum embryos were observed along with initial evidence for permanent transformation of proliferating embryo cells. Use of PEG-stimulated Cas9 and sgRNA gene uptake into rice protoplasts provided an Agrobacterium-independent method for gene delivery into plant cells and allowed a clear demonstration that mutagenesis caused by the Cas9/sgRNA complex occurs within the plant cell, free of any bacterial cell involvement.

Methods, strategies and gene constructs developed in this study should be applicable to a wide range of higher plants. Indeed, successful development of the Cas9/sgRNA system for targeted gene modification and genome editing holds significant promise for advancing fundamental knowledge of plant biology as well as for creating crop plants with valuable new agronomic, nutritional and novel traits for the benefit of farmers and consumers.

During the time the manuscript for this publication was under review, three articles appeared providing evidence for successful expression of the Cas9/sgRNA system in Arabidopsis and tobacco (46, 47) and in rice and wheat (48).

## SUPPLEMENTARY DATA

Supplementary Data are available at NAR Online.

## FUNDING

National Science Foundation (NSF) [MCB-0952533 and EPSCoR-1004094 to D.P.W.]; Department of Energy [DOE DE-EE0001052 and DOE CAB-COMM DOE DE-EE0003373 to D.P.W.]; Iowa State University Plant Science Institute Innovation Grant (to B.Y). Funding for open access charge: NSF.

*Conflict of interest statement*. None declared.

## Supplementary Material

Supplementary Data

## References

[1] Smithies O, Gregg RG, Boggs SS, Doralewski MA, Kucherlapati RS (1985). Insertion of DNA sequences into the human chromosomal beta-globin locus by homologous recombination. Nature.

[2] Capecchi MR, Thomas KR, Folger KR (1986). High frequency targeting of genes to specific sites in the mammalian genome. Cell.

[3] Capecchi MR (2001). Generating mice with targeted mutations. Nat. Med..

[4] Domínguez-López S, Howell R, Gobbi G (2012). Characterization of serotonin neurotransmission in knockout mice: implications for major depression. Rev. Neurosci..

[5] van der Weyden L, Adams DJ (2013). Cancer of mice and men: old twists and new tails. J. Pathol..

[6] Blanpain C (2013). Tracing the cellular origin of cancer. Nat. Cell Biol..

[7] Conrad M, Schick J, Angeli JP (2013). Glutathione and thioredoxin dependent systems in neurodegenerative disease: what can be learned from reverse genetics in mice. Neurochem. Int..

[8] Liu Y, Meyer C, Xu C, Weng H, Hellerbrand C, ten Dijke P, Dooley S (2013). Animal models of chronic liver diseases. Am. J. Physiol. Gastrointest. Liver Physiol..

[9] Wyman C, Kanaar R (2006). DNA double-strand break repair: all’s well that ends well. Annu. Rev. Genet..

[10] Beerli RR, Barbas CF (2002). Engineering polydactyl zincfinger transcription factors. Nat. Biotechnol..

[11] Moehle EA, Rock JM, Lee YL, Jouvenot Y, DeKelver RC, Gregory PD, Urnov FD, Holmes MC (2007). Targeted gene addition into a specified location in the human genome using designed zinc finger nucleases. Proc. Natl Acad. Sci. USA.

[12] Chen F, Pruett-Miller SM, Huang Y, Gjoka M, Duda K, Taunton J, Collingwood TN, Frodin M, Davis GD (2011). High-frequency genome editing using ssDNA oligonucleotides with zinc-finger nucleases. Nat. Methods.

[13] Gaj T, Gersbach CA, Barbas CF (2013). ZFN, TALEN, and CRISPR/Cas-based methods for genome engineering. Trends Biotechnol.

[14] Chen K, Gao C (2013). TALENs: Customizable Molecular DNA Scissors for Genome Engineering of Plants. J. Genet. Genomics..

[15] Boch J, Scholze H, Schornack S, Landgraf A, Hahn S, Kay S, Lahaye T, Nickstadt A, Bonas U (2009). Breaking the code of DNA binding specificity of TAL-type III effectors. Science.

[16] Moscou MJ, Bogdanove AJ (2009). A simple cipher governs DNA recognition by TAL effectors. Science.

[17] Li T, Huang S, Zhao X, Wright DA, Carpenter S, Spalding MH, Weeks DP, Yang B (2011). Modularly assembled designer TAL effector nucleases for targeted gene knockout and gene replacement in eukaryotes. Nucleic Acids Res..

[18] Zhang F, Cong L, Lodato S, Kosuri S, Church GM, Arlotta P (2011). Efficient construction of sequence-specific TAL effectors for modulating mammalian transcription. Nat. Biotechnol..

[19] Briggs AW, Rios X, Chari R, Yang L, Zhang F, Mali P, Church GM (2012). Iterative capped assembly: rapid and scalable synthesis of repeat-module DNA such as TAL effectors from individual monomers. Nucleic Acids Res..

[20] Schmid-Burgk JL, Schmidt T, Kaiser V, Höning K, Hornung V (2013). A ligation-independent cloning technique for high-throughput assembly of transcription activator-like effector genes. Nat. Biotechnol..

[21] Mussolino C, Morbitzer R, Lütge F, Dannemann N, Lahaye T, Cathomen T (2011). A novel TALE nuclease scaffold enables high genome editing activity in combination with low toxicity. Nucleic Acids Res..

[22] Carlson DF, Tan W, Lillico SG, Stverakova D, Proudfoot C, Christian M, Voytas DF, Long CR, Whitelaw CB, Fahrenkrug SC (2012). Efficient TALEN-mediated gene knockout in livestock. Proc. Natl Acad. Sci. USA.

[23] Li T, Liu B, Spalding MH, Weeks DP, Yang B (2012). High-efficiency TALEN-based gene editing produces disease-resistant rice. Nat. Biotechnol..

[24] Zu Y, Tong X, Wang Z, Liu D, Pan R, Li Z, Hu Y, Luo Z, Huang P, Wu Q (2013). TALEN-mediated precise genome modification by homologous recombination in zebrafish. Nat. Methods.

[25] Xiao A, Wang Z, Hu Y, Wu Y, Luo Z, Yang Z, Zu Y, Li W, Huang P, Tong X (2013). Chromosomal deletions and inversions mediated by TALENs and CRISPR/Cas in zebrafish. Nucleic Acids Res..

[26] Wiedenheft B, Sternberg SH, Doudna JA (2012). RNA-guided genetic silencing systems in bacteria and archaea. Nature.

[27] Jinek M, Chylinski K, Fonfara I, Hauer M, Doudna JA, Charpentier E (2012). A programmable dual-RNA-guided DNA endonuclease in adaptive bacterial immunity. Science.

[28] Cong L, Ran FA, Cox D, Lin S, Barretto R, Habib N, Hsu PD, Wu X, Jiang W, Marraffini LA (2013). Multiplex genome engineering using CRISPR/Cas systems. Science.

[29] Mali P, Yang L, Esvelt KM, Aach J, Guell M, DiCarlo JE, Norville JE, Church GM (2013). RNA-guided human genome engineering via Cas9. Science.

[30] Wang H, Yang H, Shivalila CS, Dawlaty MM, Cheng AW, Zhang F, Jaenisch R (2013). One-step generation of mice carrying mutations in multiple genes by CRISPR/Cas-mediated genome engineering. Cell.

[31] Fu Y, Foden JA, Khayter C, Maeder ML, Reyon D, Joung JK, Sander JD (2013). High-frequency off-target mutagenesis induced by CRISPR-Cas nucleases in human cells. Nat. Biotechnol..

[32] Qi LS, Larson MH, Gilbert LA, Doudna JA, Weissman JS, Arkin AP, Lim WA (2013). Repurposing CRISPR as an RNA-guided platform for sequence-specific control of gene expression. Cell.

[33] DiCarlo JE, Norville JE, Mali P, Rios X, Aach J, Church GM (2013). Genome engineering in Saccharomyces cerevisiae using CRISPR-Cas systems. Nucleic Acids Res..

[34] Hwang WY, Fu Y, Reyon D, Maeder ML, Tsai SQ, Sander JD, Peterson RT, Yeh JR, Joung JK (2013). Efficient genome editing in zebrafish using a CRISPR-Cas system. Nat. Biotechnol..

[35] Xiao A, Wang Z, Hu Y, Wu Y, Luo Z, Yang Z, Zu Y, Li W, Huang P, Tong X (2013). Chromosomal deletions and inversions mediated by TALENs and CRISPR/Cas in zebrafish. Nucleic Acids Res..

[36] Chang N, Sun C, Gao L, Zhu D, Xu X, Zhu X, Xiong JW, Xi JJ (2013). Genome editing with RNA-guided Cas9 nuclease in zebrafish embryos. Cell Res..

[37] Gratz SJ, Cummings AM, Nguyen JN, Hamm DC, Donohue LK, Harrison MM, Wildonger J, O'Connor-Giles KM (2013). Genome engineering of Drosophila with the CRISPR RNA-guided Cas9 nuclease. Genetics.

[38] Cho SW, Kim S, Kim JM, Kim JS (2013). Targeted genome engineering in human cells with the Cas9 RNA-guided endonuclease. Nat. Biotechnol..

[39] Tsuda K, Qi Y, Nguyen le V, Bethke G, Tsuda Y, Glazebrook J, Katagiri F (2012). An efficient Agrobacterium-mediated transient transformation of Arabidopsis. Plant J..

[40] Christensen AH, Quail PH (1996). Ubiquitin promoter-based vectors for high-level expression of selectable and/or screenable marker genes in monocotyledonous plants. Transgenic Res..

[41] McElroy D, Zhang W, Cao J, Wu R (1990). Isolation of an efficient actin promoter for use in rice transformation. Plant Cell.

[42] Howe A, Sato S, Dweikat I, Fromm M, Clemente T (2006). Rapid and reproducible Agrobacterium-mediated transformation of sorghum. Plant Cell Rep..

[43] Zhang Y, Su J, Duan S, Ao Y, Dai J, Liu J, Wang P, Li Y, Liu B, Feng D (2011). A highly efficient rice green tissue protoplast system for transient gene expression and studying light/chloroplast-related processes. Plant Methods.

[44] Porebski S, Bailey G, Baum BR (1997). Modification of a CTAB DNA extraction protocol for plants containing high polysaccharide and polyphenol components. Plant Mol. Biol. Rep..

[45] Shan Q, Wang Y, Chen K, Liang Z, Li J, Zhang Y, Zhang K, Liu J, Voytas DF, Zheng X (2013). Rapid and efficient gene modification in rice and brachypodium using TALENs. Mol. Plant.

[46] Li J, Norville JE, Aach J, McCormack M, Zhang D, Bush J, Church GM, Sheen J (2013). Multiplex and homologousrecombination–mediated genome editing in Arabidopsis and Nicotiana benthamiana using guide RNA and Cas9. Nat. Biotechnol..

[47] Nekrasov V, Staskawicz B, Weigel D, Jones JD, Kamoun S (2013). Targeted mutagenesis in the model plant Nicotiana benthamiana using Cas9 RNA-guided endonuclease. Nat. Biotechnol..

[48] Shan Q, Wang Y, Li J, Zhang Y, Chen K, Liang Z, Zhang K, Liu J, Xi J, Qiu J (2013). Targeted genome modification of crop plants using a CRISPR-Cas. system. Nat. Biotechnol..

