# Supplementary material for: Demonstration of CRISPR/Cas9/sgRNA-mediated targeted gene modification in Arabidopsis, tobacco, sorghum and rice
Source: Nucleic Acids Res. 2013 Aug 31;41(20):e188. doi: 10.1093/nar/gkt780 (PMC3814374; doi:10.1093/nar/gkt780)
Supplement: Supplementary Data [file supp_41_20_e188__index.html]

Demonstration of CRISPR/Cas9/sgRNA-mediated targeted gene modification in Arabidopsis, tobacco, sorghum and rice — Demonstration of CRISPR/Cas9/sgRNA-mediated targeted gene modification in Arabidopsis, tobacco, sorghum and rice — Supplementary Data 

# Demonstration of CRISPR/Cas9/sgRNA-mediated targeted gene modification in Arabidopsis, tobacco, sorghum and rice

## Supplementary Data

files

**Files in this Data Supplement:**

- Supplementary Data - docx file
