# Supplementary material for: Demonstration of CRISPR/Cas9/sgRNA-mediated targeted gene modification in Arabidopsis, tobacco, sorghum and rice
Source: Nucleic Acids Res. 2013 Aug 31;41(20):e188. doi: 10.1093/nar/gkt780 (PMC3814374; doi:10.1093/nar/gkt780)
Supplement: Supplementary Data [file supp_gkt780_nar-02264-met-h-2013-File002.docx]

**SUPPORTING INFORMATION**

**Demonstration of CRISPR/Cas9/sgRNA-mediated targeted gene modification in Arabidopsis, tobacco, sorghum and rice**

**Wenzhi Jiang, Huanbin Zhou, Honghao Bi, Michael Fromm, Bing Yang and Donald P. Weeks**

**A. Arabidopsis/tobacco sgRNA gene transcript.** Green: 20bp target sequence; Blue: guide sgRNA scaffold.

**GCGCTTCAAGGTGCACATGGGTTTTAGAGCTAGAAATAGCAAGTTAAAATAAGGCTAGTCCGTTATCAACTTGAAAAAGTGGCACCGAGTCGGTGCTTTTTTTT**

**B. DNA sequence of Cas9 gene used in Arabidopsis and tobacco transformation experiments.** Blue: 2XFLAG; Red: Cas9; Purple: SV40 NLS; Black: stop codon.

GATTACAAGGACGATGATGACAAGAAAGACTATAAAGATGACGATGATAAGCATATGGACAAGAAGTACAGCATCGGCCTGGACATCGGCACGAACTCGGTGGGCTGGGCGGTGATCACGGACGAGTACAAGGTGCCCTCCAAGAAGTTCAAGGTGCTGGGCAACACCGACCGCCACTCGATCAAGAAGAACCTGATCGGCGCCCTGCTGTTCGACTCCGGCGAGACCGCCGAGGCGACGCGCCTGAAGCGCACCGCGCGTCGCCGCTACACGCGTCGCAAGAACCGCATCTGCTACCTGCAGGAGATCTTCAGCAACGAGATGGCCAAGGTGGACGACTCGTTCTTCCACCGCCTGGAGGAGTCCTTCCTGGTGGAGGAAGACAAGAAGCACGAGCGCCACCCCATCTTCGGCAACATCGTGGACGAGGTGGCCTACCACGAGAAGTACCCGACGATCTACCACCTGCGCAAGAAGCTGGTGGACAGCACCGACAAGGCGGACCTGCGCCTGATCTACCTGGCCCTGGCGCACATGATCAAGTTCCGCGGCCACTTCCTGATCGAGGGCGACCTGAACCCCGACAACTCGGACGTGGACAAGCTGTTCATCCAGCTGGTGCAGACCTACAACCAGCTGTTCGAGGAGAACCCGATCAACGCCTCCGGCGTGGACGCCAAGGCGATCCTGAGCGCGCGCCTGTCCAAGAGCCGTCGCCTGGAGAACCTGATCGCCCAGCTGCCCGGCGAGAAGAAGAACGGCCTGTTCGGCAACCTGATCGCGCTGTCGCTGGGCCTGACGCCGAACTTCAAGTCCAACTTCGACCTGGCCGAGGACGCGAAGCTGCAGCTGAGCAAGGACACCTACGACGACGACCTGGACAACCTGCTGGCCCAGATCGGCGACCAGTACGCGGACCTGTTCCTGGCCGCGAAGAACCTGTCGGACGCCATCCTGCTGTCCGACATCCTGCGCGTGAACACCGAGATCACGAAGGCCCCCCTGTCGGCGTCCATGATCAAGCGCTACGACGAGCACCACCAGGACCTGACCCTGCTGAAGGCGCTGGTGCGCCAGCAGCTGCCGGAGAAGTACAAGGAGATCTTCTTCGACCAGAGCAAGAACGGCTACGCCGGCTACATCGACGGCGGCGCGTCGCAAGAGGAGTTCTACAAGTTCATCAAGCCCATCCTGGAGAAGATGGACGGCACGGAGGAGCTGCTGGTGAAGCTGAACCGCGAGGACCTGCTGCGCAAGCAGCGCACCTTCGACAACGGCAGCATCCCCCACCAGATCCACCTGGGCGAGCTGCACGCCATCCTGCGTCGCCAAGAGGACTTCTACCCGTTCCTGAAGGACAACCGCGAGAAGATCGAGAAGATCCTGACGTTCCGCATCCCCTACTACGTGGGCCCGCTGGCCCGCGGCAACAGCCGCTTCGCGTGGATGACCCGCAAGTCGGAGGAGACCATCACGCCCTGGAACTTCGAGGAAGTGGTGGACAAGGGCGCCAGCGCGCAGTCGTTCATCGAGCGCATGACCAACTTCGACAAGAACCTGCCCAACGAGAAGGTGCTGCCGAAGCACTCCCTGCTGTACGAGTACTTCACCGTGTACAACGAGCTGACGAAGGTGAAGTACGTGACCGAGGGCATGCGCAAGCCCGCCTTCCTGAGCGGCGAGCAGAAGAAGGCGATCGTGGACCTGCTGTTCAAGACCAACCGCAAGGTGACGGTGAAGCAGCTGAAAGAGGACTACTTCAAGAAGATCGAGTGCTTCGACAGCGTGGAGATCTCGGGCGTGGAGGACCGCTTCAACGCCAGCCTGGGCACCTACCACGACCTGCTGAAGATCATCAAGGACAAGGACTTCCTGGACAACGAGGAGAACGAGGACATCCTGGAGGACATCGTGCTGACCCTGACGCTGTTCGAGGACCGCGAGATGATCGAGGAGCGCCTGAAGACGTACGCCCACCTGTTCGACGACAAGGTGATGAAGCAGCTGAAGCGTCGCCGCTACACCGGCTGGGGCCGCCTGAGCCGCAAGCTGATCAACGGCATCCGCGACAAGCAGTCCGGCAAGACCATCCTGGACTTCCTGAAGAGCGACGGCTTCGCGAACCGCAACTTCATGCAGCTGATCCACGACGACTCGCTGACCTTCAAAGAGGACATCCAGAAGGCCCAGGTGTCGGGCCAGGGCGACTCCCTGCACGAGCACATCGCCAACCTGGCGGGCTCCCCCGCGATCAAGAAGGGCATCCTGCAGACCGTGAAGGTGGTGGACGAGCTGGTGAAGGTGATGGGCCGCCACAAGCCGGAGAACATCGTGATCGAGATGGCCCGCGAGAACCAGACCACGCAGAAGGGCCAGAAGAACAGCCGCGAGCGCATGAAGCGCATCGAGGAAGGCATCAAGGAGCTGGGCTCGCAGATCCTGAAGGAGCACCCCGTGGAGAACACCCAGCTGCAGAACGAGAAGCTGTACCTGTACTACCTGCAGAACGGCCGCGACATGTACGTGGACCAGGAGCTGGACATCAACCGCCTGTCCGACTACGACGTGGACCACATCGTGCCCCAGAGCTTCCTGAAGGACGACTCGATCGACAACAAGGTGCTGACCCGCAGCGACAAGAACCGCGGCAAGAGCGACAACGTGCCGTCGGAGGAAGTGGTGAAGAAGATGAAGAACTACTGGCGCCAGCTGCTGAACGCCAAGCTGATCACGCAGCGCAAGTTCGACAACCTGACCAAGGCCGAGCGCGGTGGCCTGTCGGAGCTGGACAAGGCGGGCTTCATCAAGCGCCAGCTGGTGGAGACCCGCCAGATCACGAAGCACGTGGCGCAGATCCTGGACTCCCGCATGAACACGAAGTACGACGAGAACGACAAGCTGATCCGCGAGGTGAAGGTGATCACCCTGAAGTCCAAGCTGGTCAGCGACTTCCGCAAGGACTTCCAGTTCTACAAGGTGCGCGAGATCAACAACTACCACCACGCCCACGACGCGTACCTGAACGCCGTGGTGGGCACCGCGCTGATCAAGAAGTACCCCAAGCTGGAGAGCGAGTTCGTGTACGGCGACTACAAGGTGTACGACGTGCGCAAGATGATCGCCAAGTCGGAGCAGGAGATCGGCAAGGCCACCGCGAAGTACTTCTTCTACTCCAACATCATGAACTTCTTCAAGACCGAGATCACGCTGGCCAACGGCGAGATCCGCAAGCGCCCGCTGATCGAGACCAACGGCGAGACGGGCGAGATCGTGTGGGACAAGGGCCGCGACTTCGCGACCGTGCGCAAGGTGCTGAGCATGCCCCAGGTGAACATCGTGAAGAAGACCGAGGTGCAGACGGGCGGCTTCTCCAAGGAGAGCATCCTGCCGAAGCGCAACTCGGACAAGCTGATCGCCCGCAAGAAGGACTGGGACCCCAAGAAGTACGGCGGCTTCGACTCCCCGACCGTGGCCTACAGCGTGCTGGTGGTGGCGAAGGTGGAGAAGGGCAAGTCCAAGAAGCTGAAGAGCGTGAAGGAGCTGCTGGGCATCACCATCATGGAGCGCAGCTCGTTCGAGAAGAACCCCATCGACTTCCTGGAGGCCAAGGGCTACAAAGAGGTGAAGAAGGACCTGATCATCAAGCTGCCGAAGTACTCGCTGTTCGAGCTGGAGAACGGCCGCAAGCGCATGCTGGCCTCCGCGGGCGAGCTGCAGAAGGGCAACGAGCTGGCCCTGCCCAGCAAGTACGTGAACTTCCTGTACCTGGCGTCCCACTACGAGAAGCTGAAGGGCTCGCCGGAGGACAACGAGCAGAAGCAGCTGTTCGTGGAGCAGCACAAGCACTACCTGGACGAGATCATCGAGCAGATCTCGGAGTTCTCCAAGCGCGTGATCCTGGCCGACGCGAACCTGGACAAGGTGCTGAGCGCCTACAACAAGCACCGCGACAAGCCCATCCGCGAGCAGGCGGAGAACATCATCCACCTGTTCACCCTGACGAACCTGGGCGCCCCGGCCGCGTTCAAGTACTTCGACACCACGATCGACCGCAAGCGCTACACCTCCACGAAAGAGGTGCTGGACGCGACCCTGATCCACCAGAGCATCACCGGCCTGTACGAGACGCGCATCGACCTGAGCCAGCTGGGCGGCGACTCCCGCGCGGACCCGAAGAAGAAGCGCAAGGTG**TAA**

**C. DNA sequence of nonfunctional, mutant GFP reporter gene sequence used in Arabidopsis and tobacco transformation experiments.** Red: 20bp Target; Blue: PAM; Black GFP

GCGCTTCAAGGTGCACATGGAGGACTAGTAAAGGAGAAGAACTTTTCACTGGAGTTGTCCCAATTCTTGTTGAATTAGATGGTGATGTTAATGGGCACAAATTTTCTGTCAGTGGAGAGGGTGAAGGTGATGCAACATACGGAAAACTTACCCTTAAATTTATTTGCACTACTGGAAAACTACCTGTTCCGTGGCCAACACTTGTCACTACTTTCTCTTATGGTGTTCAATGCTTTTCAAGATACCCAGATCATATGAAGCGGCACGACTTCTTCAAGAGCGCCATGCCTGAGGGATACGTGCAGGAGAGGACCATCTTCTTCAAGGACGACGGGAACTACAAGACACGTGCTGAAGTCAAGTTTGAGGGAGACACCCTCGTCAACAGGATCGAGCTTAAGGGAATCGATTTCAAGGAGGACGGAAACATCCTCGGCCACAAGTTGGAATACAACTACAACTCCCACAACGTATACATCATGGCCGACAAGCAAAAGAACGGCATCAAAGCCAACTTCAAGACCCGCCACAACATCGAAGACGGCGGCGTGCAACTCGCTGATCATTATCAACAAAATACTCCAATTGGCGATGGCCCTGTCCTTTTACCAGACAACCATTACCTGTCCACACAATCTGCCCTTTCGAAAGATCCCAACGAAAAGAGAGACCACATGGTCCTTCTTGAGTTTGTAACAGCTGCTGGGATTACACATGGCATGGATGAACTATACAAAGCTAGCCACCACCACCACCACCACGTGTGA

**D. Sequences of primers employed for PCR amplification** of the DNA region 125 bp upstream and 125 bp downstream of the 20bp Cas9/sgRNA target site in the GFP reporter gene used in Arabidopsis and tobacco experiments.

Upstream forward: 5’-TTTTTGAGCTCGATATCTCCACTGACGTAAGG Downstream reverse: 5’-TTTTTGGATCCTCCGTATGTTGCATCACCTTCAC

**E. DNA sequence of the T-DNA region (from LB to RB) in the pCAMBIA Cas9 gene + sgRNA gene vector**

TGGCAGGATATATTGTGGTGTAAACAAATTGACGCTTAGACAACTTAATAACACATTGCGGACGTTTTTAATGTACTGAATTAACGCCGAATTAATTCGGGGGATCTGGATTTTAGTACTGGATTTTGGTTTTAGGAATTAGAAATTTTATTGATAGAAGTATTTTACAAATACAAATACATACTAAGGGTTTCTTATATGCTCAACACATGAGCGAAACCCTATAGGAACCCTAATTCCCTTATCTGGGAACTACTCACACATTATTATGGAGAAACTCGAGCTTGTCGATCGACAGATCCGGTCGGCATCTACTCTATTTCTTTGCCCTCGGACGAGTGCTGGGGCGTCGGTTTCCACTATCGGCGAGTACTTCTACACAGCCATCGGTCCAGACGGCCGCGCTTCTGCGGGCGATTTGTGTACGCCCGACAGTCCCGGCTCCGGATCGGACGATTGCGTCGCATCGACCCTGCGCCCAAGCTGCATCATCGAAATTGCCGTCAACCAAGCTCTGATAGAGTTGGTCAAGACCAATGCGGAGCATATACGCCCGGAGTCGTGGCGATCCTGCAAGCTCCGGATGCCTCCGCTCGAAGTAGCGCGTCTGCTGCTCCATACAAGCCAACCACGGCCTCCAGAAGAAGATGTTGGCGACCTCGTATTGGGAATCCCCGAACATCGCCTCGCTCCAGTCAATGACCGCTGTTATGCGGCCATTGTCCGTCAGGACATTGTTGGAGCCGAAATCCGCGTGCACGAGGTGCCGGACTTCGGGGCAGTCCTCGGCCCAAAGCATCAGCTCATCGAGAGCCTGCGCGACGGACGCACTGACGGTGTCGTCCATCACAGTTTGCCAGTGATACACATGGGGATCAGCAATCGCGCATATGAAATCACGCCATGTAGTGTATTGACCGATTCCTTGCGGTCCGAATGGGCCGAACCCGCTCGTCTGGCTAAGATCGGCCGCAGCGATCGCATCCATAGCCTCCGCGACCGGTTGTAGAACAGCGGGCAGTTCGGTTTCAGGCAGGTCTTGCAACGTGACACCCTGTGCACGGCGGGAGATGCAATAGGTCAGGCTCTCGCTAAACTCCCCAATGTCAAGCACTTCCGGAATCGGGAGCGCGGCCGATGCAAAGTGCCGATAAACATAACGATCTTTGTAGAAACCATCGGCGCAGCTATTTACCCGCAGGACATATCCACGCCCTCCTACATCGAAGCTGAAAGCACGAGATTCTTCGCCCTCCGAGAGCTGCATCAGGTCGGAGACGCTGTCGAACTTTTCGATCAGAAACTTCTCGACAGACGTCGCGGTGAGTTCAGGCTTTTTCATATCTCATTGCCCCCCGGGATCTGCGAAAGCTCGAGAGAGATAGATTTGTAGAGAGAGACTGGTGATTTCAGCGTGTCCTCTCCAAATGAAATGAACTTCCTTATATAGAGGAAGGTCTTGCGAAGGATAGTGGGATTGTGCGTCATCCCTTACGTCAGTGGAGATATCACATCAATCCACTTGCTTTGAAGACGTGGTTGGAACGTCTTCTTTTTCCACGATGCTCCTCGTGGGTGGGGGTCCATCTTTGGGACCACTGTCGGCAGAGGCATCTTGAACGATAGCCTTTCCTTTATCGCAATGATGGCATTTGTAGGTGCCACCTTCCTTTTCTACTGTCCTTTTGATGAAGTGACAGATAGCTGGGCAATGGAATCCGAGGAGGTTTCCCGATATTACCCTTTGTTGAAAAGTCTCAATAGCCCTTTGGTCTTCTGAGACTGTATCTTTGATATTCTTGGAGTAGACGAGAGTGTCGTGCTCCACCATGTTATCACATCAATCCACTTGCTTTGAAGACGTGGTTGGAACGTCTTCTTTTTCCACGATGCTCCTCGTGGGTGGGGGTCCATCTTTGGGACCACTGTCGGCAGAGGCATCTTGAACGATAGCCTTTCCTTTATCGCAATGATGGCATTTGTAGGTGCCACCTTCCTTTTCTACTGTCCTTTTGATGAAGTGACAGATAGCTGGGCAATGGAATCCGAGGAGGTTTCCCGATATTACCCTTTGTTGAAAAGTCTCAATAGCCCTTTGGTCTTCTGAGACTGTATCTTTGATATTCTTGGAGTAGACGAGAGTGTCGTGCTCCACCATGTTGGCAAGCTGCTCTAGCCAATACGCAAACCGCCTCTCCCCGCGCGTTGGCCGATTCATTAATGCAGCTGGCACGACAGGTTTCCCGACTGGAAAGCGGGCAGTGAGCGCAACGCAATTAATGTGAGTTAGCTCACTCATTAGGCACCCCAGGCTTTACACTTTATGCTTCCGGCTCGTATGTTGTGTGGAATTGTGAGCGGATAACAATTTCACACAGGAAACAGCTATGACATGATTACGAATTGAGCTTGGCACTGGCCGTCGTTTTACAACGTCGTGACTGGGAAAACCCTGGCGTTACCCAACTTAATCGCCTTGCAGCACATCCCCCTTTCGCCAGCTGGCGTAATAGCGAAGAGGCCCGCACCGATCGCCCTTCCCAACAGTTGCGCAGCCTGAATGGCGAATGCTAGAGCAGCTTGAGCTTGGATCAGATTGTCGTTTCCCGCCTTCAGTTTAGCTTCATGGAGTCAAAGATTCAAATAGAGGACCTAACAGAACTCGCCGTAAAGACTGGCGAACAGTTCATACAGAGTCTCTTACGACTCAATGACAAGAAGAAAATCTTCGTCAACATGGTGGAGCACGACACACTTGTCTACTCCAAAAATATCAAAGATACAGTCTCAGAAGACCAAAGGGCAATTGAGACTTTTCAACAAAGGGTAATATCCGGAAACCTCCTCGGATTCCATTGCCCAGCTATCTGTCACTTTATTGTGAAGATAGTGGAAAAGGAAGGTGGCTCCTACAAATGCCATCATTGCGATAAAGGAAAGGCCATCGTTGAAGATGCCTCTGCCGACAGTGGTCCCAAAGATGGACCCCCACCCACGAGGAGCATCGTGGAAAAAGAAGACGTTCCAACCACGTCTTCAAAGCAAGTGGATTGATGTGATATCTCCACTGACGTAAGGGATGACGCACAATCCCACTATCCTTCGCAAGACCCTTCCTCTATATAAGGAAGTTCATTTCATTTGGAGAGAACACGGGGGACTCTTGACCATGGTAGATCTGACTAGTGATTACAAGGACGATGATGACAAGAAAGACTATAAAGATGACGATGATAAGCATATGGACAAGAAGTACAGCATCGGCCTGGACATCGGCACGAACTCGGTGGGCTGGGCGGTGATCACGGACGAGTACAAGGTGCCCTCCAAGAAGTTCAAGGTGCTGGGCAACACCGACCGCCACTCGATCAAGAAGAACCTGATCGGCGCCCTGCTGTTCGACTCCGGCGAGACCGCCGAGGCGACGCGCCTGAAGCGCACCGCGCGTCGCCGCTACACGCGTCGCAAGAACCGCATCTGCTACCTGCAGGAGATCTTCAGCAACGAGATGGCCAAGGTGGACGACTCGTTCTTCCACCGCCTGGAGGAGTCCTTCCTGGTGGAGGAAGACAAGAAGCACGAGCGCCACCCCATCTTCGGCAACATCGTGGACGAGGTGGCCTACCACGAGAAGTACCCGACGATCTACCACCTGCGCAAGAAGCTGGTGGACAGCACCGACAAGGCGGACCTGCGCCTGATCTACCTGGCCCTGGCGCACATGATCAAGTTCCGCGGCCACTTCCTGATCGAGGGCGACCTGAACCCCGACAACTCGGACGTGGACAAGCTGTTCATCCAGCTGGTGCAGACCTACAACCAGCTGTTCGAGGAGAACCCGATCAACGCCTCCGGCGTGGACGCCAAGGCGATCCTGAGCGCGCGCCTGTCCAAGAGCCGTCGCCTGGAGAACCTGATCGCCCAGCTGCCCGGCGAGAAGAAGAACGGCCTGTTCGGCAACCTGATCGCGCTGTCGCTGGGCCTGACGCCGAACTTCAAGTCCAACTTCGACCTGGCCGAGGACGCGAAGCTGCAGCTGAGCAAGGACACCTACGACGACGACCTGGACAACCTGCTGGCCCAGATCGGCGACCAGTACGCGGACCTGTTCCTGGCCGCGAAGAACCTGTCGGACGCCATCCTGCTGTCCGACATCCTGCGCGTGAACACCGAGATCACGAAGGCCCCCCTGTCGGCGTCCATGATCAAGCGCTACGACGAGCACCACCAGGACCTGACCCTGCTGAAGGCGCTGGTGCGCCAGCAGCTGCCGGAGAAGTACAAGGAGATCTTCTTCGACCAGAGCAAGAACGGCTACGCCGGCTACATCGACGGCGGCGCGTCGCAAGAGGAGTTCTACAAGTTCATCAAGCCCATCCTGGAGAAGATGGACGGCACGGAGGAGCTGCTGGTGAAGCTGAACCGCGAGGACCTGCTGCGCAAGCAGCGCACCTTCGACAACGGCAGCATCCCCCACCAGATCCACCTGGGCGAGCTGCACGCCATCCTGCGTCGCCAAGAGGACTTCTACCCGTTCCTGAAGGACAACCGCGAGAAGATCGAGAAGATCCTGACGTTCCGCATCCCCTACTACGTGGGCCCGCTGGCCCGCGGCAACAGCCGCTTCGCGTGGATGACCCGCAAGTCGGAGGAGACCATCACGCCCTGGAACTTCGAGGAAGTGGTGGACAAGGGCGCCAGCGCGCAGTCGTTCATCGAGCGCATGACCAACTTCGACAAGAACCTGCCCAACGAGAAGGTGCTGCCGAAGCACTCCCTGCTGTACGAGTACTTCACCGTGTACAACGAGCTGACGAAGGTGAAGTACGTGACCGAGGGCATGCGCAAGCCCGCCTTCCTGAGCGGCGAGCAGAAGAAGGCGATCGTGGACCTGCTGTTCAAGACCAACCGCAAGGTGACGGTGAAGCAGCTGAAAGAGGACTACTTCAAGAAGATCGAGTGCTTCGACAGCGTGGAGATCTCGGGCGTGGAGGACCGCTTCAACGCCAGCCTGGGCACCTACCACGACCTGCTGAAGATCATCAAGGACAAGGACTTCCTGGACAACGAGGAGAACGAGGACATCCTGGAGGACATCGTGCTGACCCTGACGCTGTTCGAGGACCGCGAGATGATCGAGGAGCGCCTGAAGACGTACGCCCACCTGTTCGACGACAAGGTGATGAAGCAGCTGAAGCGTCGCCGCTACACCGGCTGGGGCCGCCTGAGCCGCAAGCTGATCAACGGCATCCGCGACAAGCAGTCCGGCAAGACCATCCTGGACTTCCTGAAGAGCGACGGCTTCGCGAACCGCAACTTCATGCAGCTGATCCACGACGACTCGCTGACCTTCAAAGAGGACATCCAGAAGGCCCAGGTGTCGGGCCAGGGCGACTCCCTGCACGAGCACATCGCCAACCTGGCGGGCTCCCCCGCGATCAAGAAGGGCATCCTGCAGACCGTGAAGGTGGTGGACGAGCTGGTGAAGGTGATGGGCCGCCACAAGCCGGAGAACATCGTGATCGAGATGGCCCGCGAGAACCAGACCACGCAGAAGGGCCAGAAGAACAGCCGCGAGCGCATGAAGCGCATCGAGGAAGGCATCAAGGAGCTGGGCTCGCAGATCCTGAAGGAGCACCCCGTGGAGAACACCCAGCTGCAGAACGAGAAGCTGTACCTGTACTACCTGCAGAACGGCCGCGACATGTACGTGGACCAGGAGCTGGACATCAACCGCCTGTCCGACTACGACGTGGACCACATCGTGCCCCAGAGCTTCCTGAAGGACGACTCGATCGACAACAAGGTGCTGACCCGCAGCGACAAGAACCGCGGCAAGAGCGACAACGTGCCGTCGGAGGAAGTGGTGAAGAAGATGAAGAACTACTGGCGCCAGCTGCTGAACGCCAAGCTGATCACGCAGCGCAAGTTCGACAACCTGACCAAGGCCGAGCGCGGTGGCCTGTCGGAGCTGGACAAGGCGGGCTTCATCAAGCGCCAGCTGGTGGAGACCCGCCAGATCACGAAGCACGTGGCGCAGATCCTGGACTCCCGCATGAACACGAAGTACGACGAGAACGACAAGCTGATCCGCGAGGTGAAGGTGATCACCCTGAAGTCCAAGCTGGTCAGCGACTTCCGCAAGGACTTCCAGTTCTACAAGGTGCGCGAGATCAACAACTACCACCACGCCCACGACGCGTACCTGAACGCCGTGGTGGGCACCGCGCTGATCAAGAAGTACCCCAAGCTGGAGAGCGAGTTCGTGTACGGCGACTACAAGGTGTACGACGTGCGCAAGATGATCGCCAAGTCGGAGCAGGAGATCGGCAAGGCCACCGCGAAGTACTTCTTCTACTCCAACATCATGAACTTCTTCAAGACCGAGATCACGCTGGCCAACGGCGAGATCCGCAAGCGCCCGCTGATCGAGACCAACGGCGAGACGGGCGAGATCGTGTGGGACAAGGGCCGCGACTTCGCGACCGTGCGCAAGGTGCTGAGCATGCCCCAGGTGAACATCGTGAAGAAGACCGAGGTGCAGACGGGCGGCTTCTCCAAGGAGAGCATCCTGCCGAAGCGCAACTCGGACAAGCTGATCGCCCGCAAGAAGGACTGGGACCCCAAGAAGTACGGCGGCTTCGACTCCCCGACCGTGGCCTACAGCGTGCTGGTGGTGGCGAAGGTGGAGAAGGGCAAGTCCAAGAAGCTGAAGAGCGTGAAGGAGCTGCTGGGCATCACCATCATGGAGCGCAGCTCGTTCGAGAAGAACCCCATCGACTTCCTGGAGGCCAAGGGCTACAAAGAGGTGAAGAAGGACCTGATCATCAAGCTGCCGAAGTACTCGCTGTTCGAGCTGGAGAACGGCCGCAAGCGCATGCTGGCCTCCGCGGGCGAGCTGCAGAAGGGCAACGAGCTGGCCCTGCCCAGCAAGTACGTGAACTTCCTGTACCTGGCGTCCCACTACGAGAAGCTGAAGGGCTCGCCGGAGGACAACGAGCAGAAGCAGCTGTTCGTGGAGCAGCACAAGCACTACCTGGACGAGATCATCGAGCAGATCTCGGAGTTCTCCAAGCGCGTGATCCTGGCCGACGCGAACCTGGACAAGGTGCTGAGCGCCTACAACAAGCACCGCGACAAGCCCATCCGCGAGCAGGCGGAGAACATCATCCACCTGTTCACCCTGACGAACCTGGGCGCCCCGGCCGCGTTCAAGTACTTCGACACCACGATCGACCGCAAGCGCTACACCTCCACGAAAGAGGTGCTGGACGCGACCCTGATCCACCAGAGCATCACCGGCCTGTACGAGACGCGCATCGACCTGAGCCAGCTGGGCGGCGACTCCCGCGCGGACCCGAAGAAGAAGCGCAAGGTGTAAGAATTAATTCGGATCCATTGGTGACCAGCTCGAATTTCCCCGATCGTTCAAACATTTGGCAATAAAGTTTCTTAAGATTGAATCCTGTTGCCGGTCTTGCGATGATTATCATATAATTTCTGTTGAATTACGTTAAGCATGTAATAATTAACATGTAATGCATGACGTTATTTATGAGATGGGTTTTTATGATTAGAGTCCCGCAATTATACATTTAATACGCGATAGAAAACAAAATATAGCGCGCAAACTAGGATAAATTATCGCGCGCGGTGTCATCTATGTTACTAGATCGGGGAGCTCGAATTCCCATGGGTCGACGTAAAGCCTGTAGAAGAGGTTTCTAGCGAACGACACGAGTTTGAGCCTCATGAAGCTTCGTTGAACAACGGAAACTCGACTTGCCTTCCGCACAATACATCATTTCTTCTTAGCTTTTTTTCTTCTTCTTCGTTCATACAGTTTTTTTTTGTTTATCAGCTTACATTTTCTTGAACCGTAGCTTTCGTTTTCTTCTTTTTAACTTTCCATTCGGAGTTTTTGTATCTTGTTTCATAGTTTGTCCCAGGATTAGAATGATTAGGCATCGAACCTTCAAGAATTTGATTGAATAAAACATCTTCATTCTTAAGATATGAAGATAATCTTCAAAAGGCCCCTGGGAATCTGAAAGAAGAGAAGCAGGCCCATTTATATGGGAAAGAACAATAGTATTTCTTATATAGGCCCATTTAAGTTGAAAACAATCTTCAAAAGTCCCACATCGCTTAGATAAGAAAACGAAGCTGAGTTTATATACAGCTAGAGTCGAAGTAGTGATTGCGCTTCAAGGTGCACATGGGTTTTAGAGCTAGAAATAGCAAGTTAAAATAAGGCTAGTCCGTTATCAACTTGAAAAAGTGGCACCGAGTCGGTGCTTTTTTTTTTTGCAAAATTTTCCAGATCGATTTCTTCTTCCTCTGTTCTTCGGCGTTCAATTTCTGGGTTTTTCTCTTCGTTTTCTGTAACTGAAACCTAAAATTTGACCTAAAAAAAATCTCAAATAATATGATTCAGTGGTTTTGTACTTTTCAGTTAGTTGAGTTTTGCAGTTCCGATGAGATAAACCAATAACTTTGCTTAGATCTAATTCATTCCGTTACACCTCTGATGGAGATGGAAGGTTCTTAATAATGATGCCATTTTTTGGGTAATAATTTTGAATTAGAATCAAGGGTATAAGATTCATAATTAACATCACTTAAGCAAAGTTCGTAATATACGACCACAGGATATAATTTTTGGTACCAATTAAACTATCAGTGTTTGACAGGATATATTGGCGGGTAAACCTAAGAGAAAAGAGCGTTTA

**F. Map of pCAMBIA binary vector containing the Cas9 gene and sgRNA gene constructs**


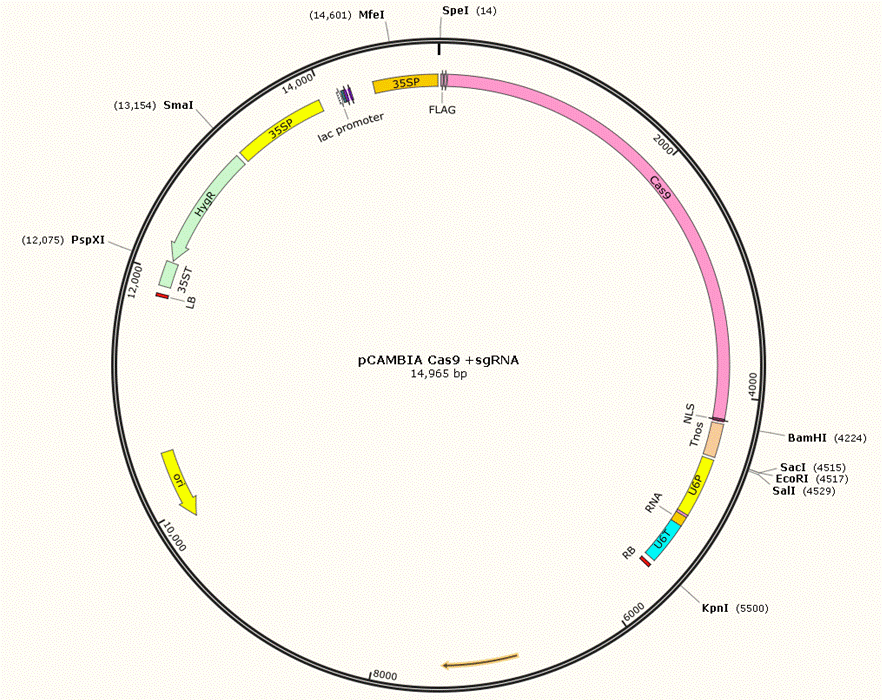


**G. DNA sequence of the T-DNA region (from LB to RB) in the pCAMBIA binary vector containing the nonfunctional mutant GFP gene**

TGGCAGGATATATTGTGGTGTAAACAAATTGACGCTTAGACAACTTAATAACACATTGCGGACGTTTTTAATGTACTGAATTAACGCCGAATTAATTCGGGGGATCTGGATTTTAGTACTGGATTTTGGTTTTAGGAATTAGAAATTTTATTGATAGAAGTATTTTACAAATACAAATACATACTAAGGGTTTCTTATATGCTCAACACATGAGCGAAACCCTATAGGAACCCTAATTCCCTTATCTGGGAACTACTCACACATTATTATGGAGAAACTCGAGCTTGTCGATCGACAGATCCGGTCGGCATCTACTCTATTTCTTTGCCCTCGGACGAGTGCTGGGGCGTCGGTTTCCACTATCGGCGAGTACTTCTACACAGCCATCGGTCCAGACGGCCGCGCTTCTGCGGGCGATTTGTGTACGCCCGACAGTCCCGGCTCCGGATCGGACGATTGCGTCGCATCGACCCTGCGCCCAAGCTGCATCATCGAAATTGCCGTCAACCAAGCTCTGATAGAGTTGGTCAAGACCAATGCGGAGCATATACGCCCGGAGTCGTGGCGATCCTGCAAGCTCCGGATGCCTCCGCTCGAAGTAGCGCGTCTGCTGCTCCATACAAGCCAACCACGGCCTCCAGAAGAAGATGTTGGCGACCTCGTATTGGGAATCCCCGAACATCGCCTCGCTCCAGTCAATGACCGCTGTTATGCGGCCATTGTCCGTCAGGACATTGTTGGAGCCGAAATCCGCGTGCACGAGGTGCCGGACTTCGGGGCAGTCCTCGGCCCAAAGCATCAGCTCATCGAGAGCCTGCGCGACGGACGCACTGACGGTGTCGTCCATCACAGTTTGCCAGTGATACACATGGGGATCAGCAATCGCGCATATGAAATCACGCCATGTAGTGTATTGACCGATTCCTTGCGGTCCGAATGGGCCGAACCCGCTCGTCTGGCTAAGATCGGCCGCAGCGATCGCATCCATAGCCTCCGCGACCGGTTGTAGAACAGCGGGCAGTTCGGTTTCAGGCAGGTCTTGCAACGTGACACCCTGTGCACGGCGGGAGATGCAATAGGTCAGGCTCTCGCTAAACTCCCCAATGTCAAGCACTTCCGGAATCGGGAGCGCGGCCGATGCAAAGTGCCGATAAACATAACGATCTTTGTAGAAACCATCGGCGCAGCTATTTACCCGCAGGACATATCCACGCCCTCCTACATCGAAGCTGAAAGCACGAGATTCTTCGCCCTCCGAGAGCTGCATCAGGTCGGAGACGCTGTCGAACTTTTCGATCAGAAACTTCTCGACAGACGTCGCGGTGAGTTCAGGCTTTTTCATATCTCATTGCCCCCCGGGATCTGCGAAAGCTCGAGAGAGATAGATTTGTAGAGAGAGACTGGTGATTTCAGCGTGTCCTCTCCAAATGAAATGAACTTCCTTATATAGAGGAAGGTCTTGCGAAGGATAGTGGGATTGTGCGTCATCCCTTACGTCAGTGGAGATATCACATCAATCCACTTGCTTTGAAGACGTGGTTGGAACGTCTTCTTTTTCCACGATGCTCCTCGTGGGTGGGGGTCCATCTTTGGGACCACTGTCGGCAGAGGCATCTTGAACGATAGCCTTTCCTTTATCGCAATGATGGCATTTGTAGGTGCCACCTTCCTTTTCTACTGTCCTTTTGATGAAGTGACAGATAGCTGGGCAATGGAATCCGAGGAGGTTTCCCGATATTACCCTTTGTTGAAAAGTCTCAATAGCCCTTTGGTCTTCTGAGACTGTATCTTTGATATTCTTGGAGTAGACGAGAGTGTCGTGCTCCACCATGTTATCACATCAATCCACTTGCTTTGAAGACGTGGTTGGAACGTCTTCTTTTTCCACGATGCTCCTCGTGGGTGGGGGTCCATCTTTGGGACCACTGTCGGCAGAGGCATCTTGAACGATAGCCTTTCCTTTATCGCAATGATGGCATTTGTAGGTGCCACCTTCCTTTTCTACTGTCCTTTTGATGAAGTGACAGATAGCTGGGCAATGGAATCCGAGGAGGTTTCCCGATATTACCCTTTGTTGAAAAGTCTCAATAGCCCTTTGGTCTTCTGAGACTGTATCTTTGATATTCTTGGAGTAGACGAGAGTGTCGTGCTCCACCATGTTGGCAAGCTGCTCTAGCCAATACGCAAACCGCCTCTCCCCGCGCGTTGGCCGATTCATTAATGCAGCTGGCACGACAGGTTTCCCGACTGGAAAGCGGGCAGTGAGCGCAACGCAATTAATGTGAGTTAGCTCACTCATTAGGCACCCCAGGCTTTACACTTTATGCTTCCGGCTCGTATGTTGTGTGGAATTGTGAGCGGATAACAATTTCACACAGGAAACAGCTATGACCATGATTACGAATTCGAGCTCGGTACCCGGGGATCCTCTAGAGTCGACCTGCAGGCATGCAAGCTTGGCACTGGCCGTCGTTTTACAACGTCGTGACTGGGAAAACCCTGGCGTTACCCAACTTAATCGCCTTGCAGCACATCCCCCTTTCGCCAGCTGGCGTAATAGCGAAGAGGCCCGCACCGATCGCCCTTCCCAACAGTTGCGCAGCCTGAATGGCGAATGCTAGAGCAGCTTGAGCTTGGATCAGATTGTCGTTTCCCGCCTTCAGTTTAGCTTCATGGAGTCAAAGATTCAAATAGAGGACCTAACAGAACTCGCCGTAAAGACTGGCGAACAGTTCATACAGAGTCTCTTACGACTCAATGACAAGAAGAAAATCTTCGTCAACATGGTGGAGCACGACACACTTGTCTACTCCAAAAATATCAAAGATACAGTCTCAGAAGACCAAAGGGCAATTGAGACTTTTCAACAAAGGGTAATATCCGGAAACCTCCTCGGATTCCATTGCCCAGCTATCTGTCACTTTATTGTGAAGATAGTGGAAAAGGAAGGTGGCTCCTACAAATGCCATCATTGCGATAAAGGAAAGGCCATCGTTGAAGATGCCTCTGCCGACAGTGGTCCCAAAGATGGACCCCCACCCACGAGGAGCATCGTGGAAAAAGAAGACGTTCCAACCACGTCTTCAAAGCAAGTGGATTGATGTGATATCTCCACTGACGTAAGGGATGACGCACAATCCCACTATCCTTCGCAAGACCCTTCCTCTATATAAGGAAGTTCATTTCATTTGGAGAGAACACGGGGGACTCTTGAC CATGGAGCGCTTCAAGGTGCACATGGAGGACTAGTAAAGGAGAAGAACTTTTCACTGGAGTTGTCCCAATTCTTGTTGAATTAGATGGTGATGTTAATGGGCACAAATTTTCTGTCAGTGGAGAGGGTGAAGGTGATGCAACATACGGAAAACTTACCCTTAAATTTATTTGCACTACTGGAAAACTACCTGTTCCGTGGCCAACACTTGTCACTACTTTCTCTTATGGTGTTCAATGCTTTTCAAGATACCCAGATCATATGAAGCGGCACGACTTCTTCAAGAGCGCCATGCCTGAGGGATACGTGCAGGAGAGGACCATCTTCTTCAAGGACGACGGGAACTACAAGACACGTGCTGAAGTCAAGTTTGAGGGAGACACCCTCGTCAACAGGATCGAGCTTAAGGGAATCGATTTCAAGGAGGACGGAAACATCCTCGGCCACAAGTTGGAATACAACTACAACTCCCACAACGTATACATCATGGCCGACAAGCAAAAGAACGGCATCAAAGCCAACTTCAAGACCCGCCACAACATCGAAGACGGCGGCGTGCAACTCGCTGATCATTATCAACAAAATACTCCAATTGGCGATGGCCCTGTCCTTTTACCAGACAACCATTACCTGTCCACACAATCTGCCCTTTCGAAAGATCCCAACGAAAAGAGAGACCACATGGTCCTTCTTGAGTTTGTAACAGCTGCTGGGATTACACATGGCATGGATGAACTATACAAAGCTAGCCACCACCACCACCACCACGTGTGAATTGGTGACCAGCTCGAATTTCCCCGATCGTTCAAACATTTGGCAATAAAGTTTCTTAAGATTGAATCCTGTTGCCGGTCTTGCGATGATTATCATATAATTTCTGTTGAATTACGTTAAGCATGTAATAATTAACATGTAATGCATGACGTTATTTATGAGATGGGTTTTTATGATTAGAGTCCCGCAATTATACATTTAATACGCGATAGAAAACAAAATATAGCGCGCAAACTAGGATAAATTATCGCGCGCGGTGTCATCTATGTTACTAGATCGGGAATTAAACTATCAGTGTTTGACAGGATATATTGGCGGGTAAACCTAAGAGAAAAGAGCGTTTA

**H. Map of the pCAMBIA binary vector containing the nonfunctional mutant GFP gene construct**


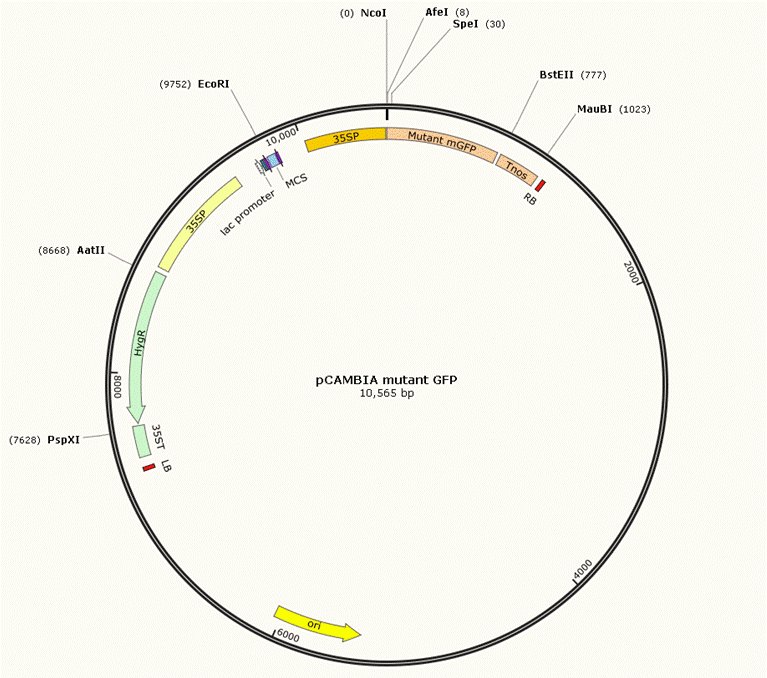


**i. Sequence of the sgRNA gene construct targeting the nonfunctional DsRed fluorescent protein gene in sorghum immature embryo cells** (Black:U6 promoter; Green: sgRNA targeting sequence; Blue: sgRNA scaffolding sequence; Red: U6 gene termination signal)

TTTGTGAAAGTTGAATTACGGCATAGCCGAAGGAATAACAGAATCGTTTCACACTTTCGTAACAAAGGTCTTCTTATCATGTTTCAGACGATGGAGGCAAGGCTGATCAAAGTGATCAAGCACATAAACGCATTTTTTTACCATGTTTCACTCC

ATAAGCGTCTGAGATTATCACAAGTCACGTCTAGTAGTTTGATGGTACACTAGTGACAATCAGTTCGTGCAGACAGAGCTCATACTTGACTACTTGAGCGATTACAGGCGAAAGTGTGAAACGCATGTGATGTGGGCTGGGAGGAGGAGAATATATACTAATGGGCCGTATCCTGATTTGGGCTGCGTCGGAAGGTGCAGCCCACGCGCGCCGTACCGCGCGGGTGGCGCTGCTACCCACTTTAGTCCGTTGGATGGGGATCCGATGGTTTGCGCGGTGGCGTTGCGGGGGATGTTTAGTACCACATCGGAAACCGAAAGACGATGGAACCAGCTTATAAACCCGCGCGCTGTAGTCAGCTT**GGGGCCACTAGGGACAGGAT**GTTTTAGAGCTAGAAATAGCAAGTTAAAATAAGGCTAGTCCGTTATCAACTTGAAAAAGTGGCACCGAGTCGGTGCTTTTTTTTT

**j. Complete DNA sequence of Cas9, sgRNA and mutant DsRED2 gene constructs between T-DNA borders for sorghum transformation experiments**

Right and left T-DNA borders are highlighted in green and the U6 transcript region of the guide RNA that targets the DsRED1 gene is in yellow.

**>Left border T-DNA fragment (green)**

GCAGGATATATTCAATTGTAAATGGCTTCATGTCCGGGAAATCTACATGGATCAGCAATGAGTATGATGGTCAATATGGAGAAAAAGAAAGAGTAATTACCAATTTTTTTTCAATTCAAAAATGTAGATGTCCGCAGCGTTATTATAAAATGAAAGTACATTTTGATAAAACGACAAATTACGATCCGTCGTATTTATAGGCGAAAGCAATAAACAAATTATTCTAATTCGGAAATCTTTATTTCGACGTGTCTACATTCACGTCCAAATGGGGGCTTAGATGAGAAACTTCACGATCGGC

**>CaMV 35S promoter/mHSP70intron/clover GFP-NptII/T35S 3’/SbfI**

TCTAGAACATGGTGGAGCACGACACTCTCGTCTACTCCAAGAATATCAAAGATACAGTCTCAGAAGACCAGAGGGCTATTGAGACTTTTCAACAAAGGGTAATATCGGGAAACCTCCTCGGATTCCATTGCCCAGCTATCTGTCACTTCATCGAAAGGACAGTAGAAAAGGAAGATGGCTTCTACAAATGCCATCATTGCGATAAAGGAAAGGCTATCGTTCAAGATGCCTCTACCGACAGTGGTCCCAAAGATGGACCCCCACCCACGAGGAACATCGTGGAAAAAgaagacgttccaaccacgtcttcaaagcaagtggattgatgtgacatctccactgacgtaagggatgacgcacaatcccactatccttcgcaag*acccttcctctatataaggaagttcatttcatttggagaggacacGCTCTCGA*CACCGTCTTCGGTACGCGCTCACTCCGCCCTCTGCCTTTGTTACTGCCACGTTTCTCTGAATGCTCTCTTGTGTGGTGATTGCTGAGAGTGGTTTAGCTGGATCTAGAATTACACTCTGAAATCGTGTTCTGCCTGTGCTGATTACTTGCCGTCCTTTGTAGCAGCAAAATATAGGGACATGGTAGTACGAAACGAAGATAGAACCTACACAGCAATACGAGAAATGTGTAATTTGGTGCTTAGCGGTATTTATTTAAGCACATGTTGGTGTTATAGGGCACTTGGATTCAGAAGTTTGCTGTTAATTTAGGCACAGGCTTCATACTACATGGGTCAATAGTATAGGGATTCATATTATAGGCGATACTATAATAATTTGTTCGTCTGCAGAGCTTATTATTTGCCAAAATTAGATATTCCTATTCTGTTTTTGTTTGTGTGCTGTTAAATTGTTAACGCCTGAAGGAATAAATATAAATGACGAAATTTTGATGTTTATCTCTGCTCCTTTATTGTGACCATAAGTCAAGATCAGATGCACTTGTTTTAAATATTGTTGTCTGAAGAAATAAGTACTGACAGTATTTTGATGCATTGATCTGCTTGTTTGTTGTAACAAAATTTAAAAATAAAGAGTTTCCTTTTTGTTGCTCTCCTTACCTCCTGATGGTATCTAGTATCTACCAACTGACACTATATTGCTTCTCTTTACATACGTATCTTGCTCGATGCCTTCTCCCTAGTGTTGACCAGTGTTACTCACATAGTCTTTGCTCATTTCATTGTAATGCAGATACCAAGCGGCTCGACACCGTCTTCGGTACGCGCTCACTCCGCCCTCTGCCTTTGTTACTGCCACGTTTCTCTGAATGCTCTCTTGTGTGGTGATTGCTGAGAGTGGTTTAGCTGGATCTAGAATTACACTCTGAAATCGTGTTCTGCCTGTGCTGATTACTTGCCGTCCTTTGTAGCAGCAAAATATAGGGACATGGTAGTACGAAACGAAGATAGAACCTACACAGCAATACGAGAAATGTGTAATTTGGTGCTTAGCGGTATTTATTTAAGCACATGTTGGTGTTATAGGGCACTTGGATTCAGAAGTTTGCTGTTAATTTAGGCACAGGCTTCATACTACATGGGTCAATAGTATAGGGATTCATATTATAGGCGATACTATAATAATTTGTTCGTCTGCAGAGCTTATTATTTGCCAAAATTAGATATTCCTATTCTGTTTTTGTTTGTGTGCTGTTAAATTGTTAACGCCTGAAGGAATAAATATAAATGACGAAATTTTGATGTTTATCTCTGCTCCTTTATTGTGACCATAAGTCAAGATCAGATGCACTTGTTTTAAATATTGTTGTCTGAAGAAATAAGTACTGACAGTATTTTGATGCATTGATCTGCTTGTTTGTTGTAACAAAATTTAAAAATAAAGAGTTTCCTTTTTGTTGCTCTCCTTACCTCCTGATGGTATCTAGTATCTACCAACTGACACTATATTGCTTCTCTTTACATACGTATCTTGCTCGATGCCTTCTCCCTAGTGTTGACCAGTGTTACTCACATAGTCTTTGCTCATTTCATTGTAATGCAGATACCAAGCGGCTCGACCCCACCACCATGGTGAGCAAGGGCGAGGAGCTGTTCACTGGGGTGGTGCCCATCCTGGTCGAGCTGGACGGCGACGTGAACGGCCACAAGTTCAGCGTCCGAGGCGAGGGCGAGGGCGACGCCACCAACGGCAAGCTGACCCTGAAGTTCATCTGCACCACCGGCAAGCTGCCCGTGCCCTGGCCCACCCTCGTGACCACCTTCGGCTACGGCGTGGCCTGCTTCAGCCGCTACCCCGACCACATGAAGCAGCACGACTTCTTCAAGTCCGCCATGCCCGAAGGCTACGTCCAGGAGCGCACCATCTCTTTCAAGGACGACGGCACCTACAAGACCCGAGCCGAGGTGAAGTTCGAGGGCGACACCCTGGTGAACCGCATCGAGCTGAAGGGCATCGACTTCAAGGAGGACGGCAACATCCTGGGGCACAAGCTGGAGTACAACTTCAACAGCCACAACGTCTACATCACGGCTGACAAGCAGAAGAACGGCATCAAGGCCAACTTCAAGATCCGCCACAACGTCGAGGACGGCAGCGTCCAGCTCGCCGACCACTACCAGCAGAACACGCCCATCGGCGACGGTCCCGTGCTGCTGCCCGACAACCACTACCTGAGCCACCAGTCCGCTCTGAGCAAGGACCCCAACGAGAAGCGCGACCACATGGTCCTGCTGGAGTTCGTCACCGCAGCTGGCATCACCCACGGCATGGACGAGCTGTACAAGCCGCGGggaggaagcAtgatcgagcaggacggcctgcacgcTggctccccAgcTgcctgggtggagaggctgttcggctacgactgggcTcagcagaccatcggctgctccgacgcTgccgtgttcaggctgtccGCAcagggcaggccAgtgctgttcgtgaagaccgacctgtccggAgccctgaacgagctCcaggacgaggcAgccaggctgtcctggctggccaccaccggAgtgccgtgcgcAgccgtgctggacgtggtgaccgaggcAggcagggactggctgctgctgggcgaggtgccAggccaggacctgctgtcctcccacctggcAccggCAgagaaggtgtccatcatggccgacgccatgaggaggctgcacaccctggacccAgccacctgcccgttcgaccaccaggccaagcacaggatcgagagggccaggaccaggatggaggcAggcctggtggaccaggacgacctggacgaggagcaccagggcctggcAccAgccgagctgttcgccaggctgaaggccaggatgccggacggcgaggacctggtggtgacccacggcgacgcctgcctgccgaacatcatggtggagaacggcaggttctccggcttcatcgactgcggcaggctgggcgtggccgaccgctaccaggacatcgccctggccaccagggacatcgccgaggagctgggAggcgagtgggcagacaggttcctggtgctgtacggcatcgcAgcAccggactcccagaggatcgccttctaccgcctgctggacgagttcttcTGAAAATCACCAGtctctctctacaaatctatctctctctatttttctcCAGAATAATGTGTGAGTAGTTCCCAGATAAGGGAATTAGGGTTCTTATAGGGTTTCGCTCATGTGTTGAGCATATAAGAAACCCTTAGTATGTATTTGTATTTGTAAAATACTTCTATCAATAAAATTTCTAATTCCTAAAACCAAAATCCAGT CCTGCAGG

**>Maize ubiquitin 1 promoter/intron**

GCATGCAAGCTGATCCACTAGAggccatggcggccgCactaggctgcagtgcagcgtgacccggtcgtgcccctctctagagataatgagcattgcatgtctaagttataaaaaattaccacatattttttttgtcacacttgtttgaagtgcagtttatctatctttatacatatatttaaactttactctacgaataatataatctatagtactacaataatatcagtgttttagagaatcatataaatgaacagttagacatggtctaaaggacaattgagtattttgacaacaggactctacagttttatctttttagtgtgcatgtgttctcctttttttttgcaaatagcttcacctatataatacttcatccattttattagtacatccatttagggtttagggttaatggtttttatagactaatttttttagtacatctattttattctattttagcctctaaattaagaaaactaaaactctattttagtttttttatttaataatttagatataaaatagaataaaataaagtgactaaaaattaaacaaataccctttaagaaattaaaaaaactaaggaaacatttttcttgtttcgagtagataatgccagcctgttaaacgccgtcgatcgacgagtctaacggacaccaaccagcgaaccagcagcgtcgcgtcgggccaagcgaagcagacggcacggcatctctgtcgctgcctctggacccctctcgagagttccgctccaccgttggacttgctccgctgtcggcatccagaaattgcgtggcggagcggcagacgtgagccggcacggcaggcggcctcctcctcctctcacggcaccggcagctacgggggattcctttcccaccgctccttcgctttcccttcctcgcccgccgtaataaatagacaccccctccacaccctctttccccaacctcgtgttgttcggagcgcacacacacacaaccagatctcccccaaatccacccgtcggcacctccgcttcaaggtacgccgctcgtcctccccccccccccctctctaccttctctagatcggcgttccggtccatggttagggcccggtagttctacttctgttcatgtttgtgttagatccgtgtttgtgttagatccgtgctgctagcgttcgtacacggatgcgacctgtacgtcagacacgttctgattgctaacttgccagtgtttctctttggggaatcctgggatggctctagccgttccgcagacgggatcgatctaggataggtatacatgttgatgtgggttttactgatgcatatacatgatggcatatgcagcatctattcatatgctctaaccttgagtacctatctattataataaacaagtatgttttataattattttgatcttgatatacttggatgatggcatatgcagcagctatatgtggatttttttagccctgccttcatacgctatttatttgcttggtactgtttcttttgtcgatgctcaccctgttgtttggtgttacttctgcag gtactagtt ggatcc

**>DsRED2 out of frame/NOS3’**

CTCGAGCAACAAACCATGGGCCAATTGACGGGGCCACTAGGGACAGGATTGGGCCTCCTCCGAGAACGTCATCACCGAGTTCATGCGCTTCAAGGTGCGCATGGAGGGCACCGTGAACGGCCACGAGTTCGAGATCGAGGGCGAGGGCGAGGGCCGCCCCTACGAGGGCCACAACACCGTGAAGCTGAAGGTGACCAAGGGCGGCCCCCTGCCCTTCGCCTGGGACATCCTGTCCCCCCAGTTCCAGTACGGCTCCAAGGTGTACGTGAAGCACCCCGCCGACATCCCCGACTACAAGAAGCTGTCCTTCCCCGAGGGCTTCAAGTGGGAGCGCGTGATGAACTTCGAGGACGGCGGCGTGGCGACCGTGACCCAGGACTCCTCCCTGCAGGACGGCTGCTTCATCTACAAGGTGAAGTTCATCGGCGTGAACTTCCCCTCCGACGGCCCCGTGATGCAGAAGAAGACCATGGGCTGGGAGGCCTCCACCGAGCGCCTGTACCCCCGCGACGGCGTGCTGAAGGGCGAGACCCACAAGGCCCTGAAGCTGAAGGACGGCGGCCACTACCTGGTGGAGTTCAAGTCCATCTACATGGCCAAGAAGCCCGTGCAGCTGCCCGGCTACTACTACGTGGACGCCAAGCTGGACATCACCTCCCACAACGAGGACTACACCATCGTGGAGCAGTACGAGCGCACCGAGGGCCGCCACCACCTGTTCCTGTGACCGCGGAATGAGCTCTGTCCAACAGTCTCAGGGTTAATGTCTATGTATCTTAAATAATGTTGTCGGCGATCGTTCAAACATTTGGCAATAAAGTTTCTTAAGATTGAATCCTGTTGCCGGTCTTGCGATGATTATCATATAATTTCTGTTGAATTACGTTAAGCATGTAATAATTAACATGTAATGCATGACGTTATTTATGAGATGGGTTTTTATGATTAGAGTCCCGCAATTATACATTTAATACGCGATAGAAAACAAAATATAGCGCGCAAACTAGGATAAATTATCGCGCGCGGTGTCATCTATGTTACTAGATC

**>Rice Act1 promoter/intron**

AAGCTTGCATGCCTGCAGGTCGAGGTCATTCATATGCTTGAGAAGAGAGTCGGGATAGTCCAAAATAAAACAAAGGTAAGATTACCTGGTCAAAAGTGAAAACATCAGTTAAAAGGTGGTATAAAGTAAAATATCGGTAATAAAAGGTGGCCCAAAGTGAAATTTACTCTTTTCTACTATTATAAAAATTGAGGATGTTTTGTCGGTACTTTGATACGTCATTTTTGTATGAATTGGTTTTTAAGTTTATTCGCGATTTGGAAATGCATATCTGTATTTGAGTCGGTTTTTAAGTTCGTTGCTTTTGTAAATACAGAGGGATTTGTATAAGAAATATCTTTAAAAAACCCATATGCTAATTTGACATAATTTTTGAGAAAAATATATATTCAGGCGAATTCCACAATGAACAATAATAAGATTAAAATAGCTTGCCCCCGTTGCAGCGATGGGTATTTTTTCTAGTAAAATAAAAGATAAACTTAGACTCAAAACATTTACAAAAACAACCCCTAAAGTCCTAAAGCCCAAAGTGCTATGCACGATCCATAGCAAGCCCAGCCCAACCCAACCCAACCCAACCCACCCCAGTGCAGCCAACTGGCAAATAGTCTCCACCCCCGGCACTATCACCGTGAGTTGTCCGCACCACCGCACGTCTCGCAGCCAAAAAAAAAAAAAGAAAGAAAAAAAAGAAAAAGAAAAACAGCAGGTGGGTCCGGGTCGTGGGGGCCGGAAAAGCGAGGAGGATCGCGAGCAGCGACGAGGCCCGGCCCTCCCTCCGCTTCCAAAGAAACGCCCCCCATCGCCACTATATACATACCCCCCCCTCTCCTCCCATCCCCCCAACCCTACCACCACCACCACCACCACCTCCTCCCCCCTCGCTGCCGGACGACGAGCTCCTCCCCCCTCCCCCTCCGCCGCCGCCGGTAACCACCCCGCCCCTCTCCTCTTTCTTTCTCCGTTTTTTTTTTCGTCTCGGTCTCGATCTTTGGCCTTGGTAGTTTGGGTGGGCGAGAGCGGCTTCGTCGCCCAGATCGGTGCGCGGGAGGGGCGGGATCTCGCGGCTGGCGTCTCCGGGCGTGAGTCGGCCCGGATCaTCGCGGGGAATGGGGCTCTCGGATGTAaATCTGCGATCCGCCGTTGTTGGGGGAGATGATGGGGGGTTTAAAATTTCCGCCATGCTAAACAAGATCAGGAAGAGGGGAAAAGGGCACTATGGTTTATATTTTTATATATTTCTGCTGCTTCGTCAGGCTTAGATGTGCTAaATCTTTCTTTCTTCTTTTTGTGGTAGAATTTGAATCCCTCAGCATTGTTCATCGGTAGTTTTTCTTTTCATGATTTGTGACAAATGCAGCCTCGTGCGGAGCTTTTTTGTAG GTAGACC

**>Synthetic Cas9**

CTCGAGggcAccATGgacaagaagtactccatcggcctggacatcggcaccaactctgtgggctgggccgtgatcaccgacgagtacaaggtgcccagcaagaaGttcaaggtgctgggcaacaccgaccggcacagcatcaagaagaacctgatcggagccctgctgttcgacagcggcgaGacCgccgaggccacccggctgaagagGaccgccagGagGagGtacaccagGcggaagaaccggatctgctaCctCcaGgagatTttcagcaacgagatggccaaggtggacgacagcttcttccacagGctggaGgagtccttcctggtggaGgaggaCaagaagcacgagcggcaccccatcttcggcaacatcgtggacgaggtggcctaccacgagaagtaccccaccatctaccacctgagGaagaaGctggtggacagcaccgacaaggccgacctgcggctgatctaCctggccctggcccacatgatcaagttccggggAcacttcctgatcgagggcgacctgaaccccgacaacagcgacgtggacaagctgttcatccagctggtCcagacctacaaccagctgttcgaggaGaaccccatcaacgccagcggcgtggacgccaaggccatcctgtctgccagGctgagcaagagcagGcggctggaGaatctgatcgcccagctgccTggcgagaagaagaaCggcctgttcggcaacctgatCgccctgagcctgggcctgacccccaacttcaagagcaacttcgacctggccgaggaCgccaaGctCcagctgagcaaggacacctacgacgacgacctggacaacctgctggcccagatcggcgaccagtacgccgacctgttCctggccgccaagaacctgtccgacgccatcctgctgagcgacatcctgagGgtgaacaccgagatcaccaaggccccActgagcgcctcCatgatcaagagGtacgacgagcaccaccaggacctgaccctgctgaaGgctctcgtgcggcagcagctgcctgagaagtacaaGgagatCttcttcgaccagagcaagaacggctacgccggctacatCgacggcggagccagccaggaGgagttctacaagttcatcaagcccatcctggaGaagatggacggcaccgaggaGctgctcgtgaagctgaacagGgaggacctgctgcggaagcagcggaccttcgacaacggcagcatcccGcaccagatccacctgggagagctgcacgccatCctgAggcggcaggaGgaCttCtacccgttcctgaaggacaaccgggaGaagatcgagaagatcctgaccttccgcatcccctactacgtgggccctctggccaggggcaacagcagGttcgcctggatgaccagGaagagcgaggaGaccatcaccccgtggaacttcgaggaGgtggtggacaagggcgcttccgcccagagcttcatcgagcggatgaccaacttcgaCaagaacctgcccaacgagaaggtgctgcccaagcacagcctgctgtacgagtacttcaccgtgtaCaacgagctgaccaaGgtgaaGtacgtgaccgagggCatgagGaagccAgccttcctgagcggcgagcagaaGaaggccatcgtggacctgctgttcaagaccaaccggaaGgtgaccgtgaagcagctgaaGgaggactacttcaagaaGatcgagtgcttcgactccgtggaGatcagcggcgtggaGgaCcggttcaacgcctccctgggcacCtaccacgaCctgctgaaGatCatcaaggacaaggacttcctggacaaCgaggaGaacgaggacatCctggaGgaCatcgtgctgaccctgacCctgttCgaggacagGgagatgatcgaggaGcggctgaaGacctaCgcccacctgttcgacgacaaGgtgatgaagcagctgaagcggcggagGtacaccggctggggcaggctgagccggaagctgatcaacggcatccgggacaagcagtccggcaagacCatcctggaCttcctgaagtccgacggcttcgccaacagGaacttcatgcaActgatccacgacgacagcctgaccttCaaGgaggacatccagaaGgcccaggtgtccggccagggcgaCagcctgcacgagcacatCgccaaCctggccggcagcccAgccatCaagaagggcatcctCcagacCgtgaaggtggtggacgagctcgtgaaGgtgatgggcAggcacaagcccgagaacatcgtgatcgaGatggccagGgagaaccagaccacccagaagggacagaagaacagccgcgagagGatgaagcggatcgaGgagggcatcaaGgagctgggcagccagatcctgaaGgaGcacccGgtggaGaacacccagctccagaacgagaagctgtacctgtactacctccagaaCggAcgggaCatgtacgtggaccaggaGctggacatcaaccggctgtccgactacgaCgtggaccaCatcgtgcctcagagcttCctgaaggacgactccatcgacaacaaggtgctgaccagGagcgacaagaacAggggcaagagcgacaacgtgccctccgaGgaggtcgtgaagaagatgaagaactactggcggcagctgctgaacgccaagctgatCacccagagGaagttcgacaaCctgaccaaggccgagCGCggAggcctgagcgaGctggaCaaggccggcttcatcaagagGcagctggtggaGacccggcagatcacCaagcacgtggcCcagatcCtggactcccggatgaacacCaagtacgacgagaaCgacaagctgatccgggaGgtgaaGgtgatcaccctgaagtccaagctggtgtccgaCttccggaaggaCttccagttCtacaaGgtgcgcgagatcaacaactaccaccacgcccacgacgcctacctgaacgccgtcgtgggCaccgccctgatcaaGaagtacccGaagctggaGagcgagttcgtgtacggcgactacaaggtgtacgacgtgcggaagatgatcgccaagagcgagcaggaGatcggcaaggcCaccgccaagtacttcttctactccaacatcatgaacttCttcaagaccgagatCaccctggccaacggcgagatccggaagcggccActgatcgagacCaacggcgaGaccggCgagatcgtgtgggaCaagggAcgggaCttCgccaccgtgcggaaGgtgctgagcatgccGcaGgtgaaCatcgtgaaGaagaccgaggtCcagacCggAggcttcagcaaGgagtcCatcctgcccaagaggaacagcgaCaagctgatcgccagGaagaaggactgggacccGaagaagtacggcggcttcgacagccccaccgtggcctaCtcCgtgctggtggtggccaaGgtggaGaagggcaagtccaagaaGctgaagagCgtgaaGgagctgctggggatcaccatcatggaGagGagcagcttcgagaagaatcccatcgacttCctggaGgccaagggctacaaGgaGgtgaaGaaggacctgatcatcaagctgccCaagtactccctgttcgagctggaGaacggccggaagagGatgctggcctcCgcAggcgaGcttcagaagggCaacgaGctAgccctgccctccaaGtaCgtgaacttcctgtacctggccagccactaCgagaagctgaagggcAGCccGgaggaCaaCgagcagaaGcagctgttCgtggaGcagcacaagcactacctggacgagatcatcgagcagatcagcgagttctccaagagGgtgatcctggccgacgctaaCctggacaaGgtgctgtccgcctacaacaagcaccgggaCaagcccatcagGgagcaggccgagaaCatcatccacctgttCaccctgaccaaCctgggagcGcctgccgccttcaagtacttCgacaccaccatcgaccggaagaggtacaccagcaccaaGgaggtgctggacgccaccctgatccaccagagcatcaccggcctgtacgagacCcggatcgacCtgtcCcagctgggaggcgacGGCAGCGGGAGCGACCCAAAgAAGAAGCGGAAGGTAGAcCCtAAgAAGAAGcgCAAgGTcTGACCGCGG

**>OCS 3’ terminator**

CTGCTTTAATGAGATATGCGAGACGCCTATGATCGCATGATATTTGCTTTCAATTCTGTTGTGCACGTTGTAAAAACCTGAGCATGTGTAGCTCAGATCCTTACCGCCGGTTTCGGTTCATTCTAATGAATATATCACCCGTTACTATCGTATTTTTATGAATAATATTCTCCGTTCAATTTACTGATTGTACCCTACTACTTATATGTACAATATTAAAATGAAAACAATATATTGTGCTGAATAGGTTTATAGCGACATCTATGATAGAGCGCCACAATAACAAACAATTGCGTTTTATTATTACAAATCCAATTTT

**> Rice U6/T2 guide sequence (DsRED2 target homology in yellow)**

CccgggTTTGTGAAAGTTGAATTACGGCATAGCCGAAGGAATAACAGAATCGTTTCACACTTTCGTAACAAAGGTCTTCTTATCATGTTTCAGACGATGGAGGCAAGGCTGATCAAAGTGATCAAGCACATAAACGCATTTTTTTACCATGTTTCACTCCATAAGCGTCTGAGATTATCACAAGTCACGTCTAGTAGTTTGATGGTACACTAGTGACAATCAGTTCGTGCAGACAGAGCTCATACTTGACTACTTGAGCGATTACAGGCGAAAGTGTGAAACGCATGTGATGTGGGCTGGGAGGAGGAGAATATATACTAATGGGCCGTATCCTGATTTGGGCTGCGTCGGAAGGTGCAGCCCACGCGCGCCGTACCGCGCGGGTGGCGCTGCTACCCACTTTAGTCCGTTGGATGGGGATCCGATGGTTTGCGCGGTGGCGTTGCGGGGGATGTTTAGTACCACATCGGAAACCGAAAGACGATGGAACCAGCTTATAAACCCGCGCGCTGTAGTCAGCTTGGGGCCACTAGGGACAGGATGTTTTAGAGCTAGAAATAGCAAGTTAAAATAAGGCTAGTCCGTTATCAACTTGAAAAAGTGGCACCGAGTCGGTGCTTTTTTTTTGCGAATTCTGGCCAGGTCCTTGCGCGTTCGGTATTTT GGTACC

**T-DNA right border (green)**

CGATCAGATTGTCGTTTCCCGCCTTCGGTTTAAACTATCAGTGTTTGACAGGATATATTGGCGGGTAAAC

**K. Sequences of primers employed for PCR amplification** **of the Cas9 gene of *Streptococcus pyogenes.***

Cas9-F1, aagatctATGGATAAGAAATACTCAATAGGCT

Cas9-R1, tcaactagtGTCACCTCCTAGCTGACTCAAATC

# L. Sequence of the rice U6 promoter/terminator construct

# ATGCAAGAACGAACTAAGCCGGACAAAAAAAAAAAGGAGCACATATACAAACCGGTTTTATTCATGAATGGTCACGATGGATGATGGGGCTCAGACTTGAGCTACGAGGCCGCAGGCGAGAGAAGCCTAGTGTGCTCTCTGCTTGTTTGGGCCGTAACGGAGGATACGGCCGACGAGCGTGTACTACCGCGCGGGATGCCGCTGGGCGCTGCGGGGGCCGTTGGATGGGGATCGGTGGGTCGCGGGAGCGTTGAGGGGAGACAGGTTTAGTACCACCTCGCCTACCGAACAATGAAGAACCCACCTTATAACCCCGCGCGCTGCCGCTTGTGTTGGGAGACCGAGGTCTCGGTTTTAGAGCTAGAAATAGCAAGTTAAAATAAGGCTAGTCCGTTTTTTTTTGAGATTTCCAACCAGGTCCCTGGAGCCCATAGTTTTCCTTGCAGATACGCCCCTCCGCCTATTCCAACTCTTACAACTCGGTCCTTTTTATACATTTCCTTGTCAATTGCACCCTCAGTCTCTACCTTTGCGGATATACCCACAAATGATCATCATTTTAGAATTCACGTTCTGGTCCCTGCGTCCAGTTTTTCCTTGCAGATATACCCATACCTCCTTACAACTCGGTCCTTCACC

**M. Sequences of primers employed for PCR amplification of the rice U6 gene.**

U6P-F1: ATGCAAGAACGAACTAAGCCGGAC

U6P-R1: TTAACTTGCTATTTCTAGCTCTAAAACCGAGACCTCGGTCTCCCAACACAAGCGGCAGCGCGCGGGGTTA

U6P-F2: TTAGAGCTAGAAATAGCAAGTTAAAATAAGGCTAGTCCGTTTTTTTTTGAGATTTCCAACCAGGTCCCTG

U6P-R2: GGTGAAGGACCGAGTTGTAAGGAGGTATG

**N. Rice codon optimized Cas9 sequence**

ATGGACTATAAGGATCACGATGGCGACTACAAGGATCATGACATTGACTATAAGGATGACGACGATAAGATGGCACCTAAGAAGAAAAGGAAAGTCGGCATTCATGGCGTTCCGGCAGCCGACAAAAAGTATAGCATCGGCCTCGATATTGGGACAAACTCTGTGGGCTGGGCGGTAATTACCGACGAGTACAAGGTGCCTAGTAAGAAATTTAAAGTGCTCGGAAACACTGACAGGCACTCTATAAAGAAGAACCTGATCGGGGCACTGCTTTTCGACTCCGGAGAGACGGCGGAGGCGACGCGTCTCAAGCGTACCGCGCGCCGCAGGTACACAAGAAGGAAGAATAGGATCTGCTACTTGCAGGAAATCTTCAGTAACGAGATGGCGAAGGTCGACGATAGTTTCTTTCATCGGTTGGAAGAATCGTTCCTCGTAGAGGAGGACAAAAAGCACGAGCGTCACCCAATATTCGGGAATATTGTTGACGAGGTTGCCTACCATGAGAAATATCCTACAATATATCACCTCCGTAAGAAGCTTGTCGATTCAACTGATAAGGCTGATCTCAGACTCATCTATCTTGCCCTCGCACATATGATTAAGTTTCGTGGCCACTTCTTGATTGAAGGCGACCTCAACCCGGACAACTCAGATGTTGACAAGCTTTTTATACAGCTCGTCCAGACATATAACCAGCTGTTTGAAGAGAATCCCATCAATGCGAGTGGGGTTGATGCTAAGGCCATTTTGTCCGCCAGGTTGTCCAAATCTCGCAGACTGGAAAACCTGATCGCACAGCTTCCCGGTGAAAAGAAAAACGGGCTCTTCGGCAATCTCATCGCACTGTCCCTCGGCCTCACCCCAAACTTCAAGTCTAACTTCGACCTGGCCGAGGATGCGAAGCTCCAGCTGTCAAAAGATACATACGACGACGATTTGGACAATCTGCTTGCGCAAATAGGCGACCAGTATGCGGACCTGTTCCTGGCTGCCAAAAATCTGTCAGATGCAATCCTCCTGTCCGATATATTGCGTGTGAACACCGAAATCACGAAGGCACCGCTTAGCGCATCCATGATCAAGAGATACGACGAGCACCATCAGGACCTCACACTCCTCAAGGCGCTTGTTCGTCAGCAGCTTCCCGAGAAATATAAGGAAATTTTTTTCGATCAAAGCAAGAATGGATATGCTGGCTATATTGACGGTGGCGCTTCGCAGGAGGAGTTCTATAAATTCATTAAGCCGATTCTGGAGAAGATGGACGGAACGGAGGAGCTCCTCGTCAAGCTTAACCGGGAAGACCTGTTGCGGAAGCAGAGGACTTTTGATAACGGCTCTATTCCGCACCAAATCCATCTGGGTGAGTTGCACGCAATCTTGAGAAGACAAGAGGATTTCTACCCGTTCCTTAAGGATAACAGAGAGAAGATAGAAAAAATACTGACCTTCAGGATACCATACTATGTGGGCCCACTGGCGCGCGGAAATAGTCGTTTCGCATGGATGACTAGAAAGTCCGAAGAAACGATCACGCCATGGAATTTTGAGGAAGTGGTCGACAAGGGCGCCTCTGCCCAGAGCTTCATCGAAAGGATGACCAATTTTGACAAAAATCTGCCTAACGAAAAGGTGCTTCCGAAGCACAGCCTGTTGTATGAATACTTCACAGTTTATAACGAGCTCACTAAGGTCAAGTACGTCACGGAGGGCATGCGTAAGCCTGCTTTCCTGTCTGGTGAACAAAAAAAGGCGATTGTGGACCTCCTTTTCAAGACGAACCGTAAAGTTACTGTGAAGCAACTGAAAGAGGATTACTTTAAGAAAATTGAGTGCTTCGACAGTGTGGAGATTTCCGGTGTCGAGGACCGGTTTAACGCCAGCCTGGGTACGTATCATGACCTGCTTAAAATTATCAAGGATAAAGATTTCCTGGATAATGAAGAGAACGAAGATATACTGGAGGACATTGTGTTGACTTTGACCCTCTTCGAGGACAGAGAGATGATTGAGGAAAGACTGAAGACCTACGCACACCTTTTTGATGACAAGGTCATGAAACAACTCAAGCGCCGGCGCTATACTGGCTGGGGCCGGCTTTCTCGCAAGCTCATCAATGGGATTCGGGATAAGCAATCAGGCAAGACAATTTTGGACTTCCTCAAATCCGACGGATTCGCAAATAGGAATTTTATGCAGCTGATACATGACGACTCTTTGACATTCAAAGAAGACATACAGAAGGCTCAGGTCTCCGGCCAAGGAGATTCTTTGCACGAGCATATCGCTAACTTGGCAGGTAGCCCCGCCATAAAAAAGGGCATTCTTCAAACGGTAAAAGTTGTTGACGAACTCGTGAAGGTTATGGGCCGTCATAAGCCGGAAAACATTGTTATTGAAATGGCTAGGGAAAATCAGACGACCCAGAAGGGACAGAAAAATAGCAGGGAGCGGATGAAGAGAATTGAAGAGGGAATTAAGGAGCTTGGATCTCAGATTCTTAAGGAGCACCCTGTGGAGAACACCCAACTTCAGAATGAAAAGCTCTACCTTTACTACCTTCAAAACGGCCGGGATATGTACGTCGATCAGGAACTTGACATTAACCGGTTGAGCGATTATGACGTTGACCATATTGTGCCCCAATCTTTCCTTAAAGACGACTCTATCGACAATAAAGTGCTGACGCGCAGCGATAAAAATCGCGGTAAGTCGGATAATGTCCCGTCGGAAGAGGTGGTTAAAAAAATGAAGAACTATTGGAGGCAACTCCTGAATGCCAAGCTGATCACTCAGAGGAAATTCGACAATCTCACCAAGGCAGAAAGGGGTGGACTTAGCGAGCTCGACAAGGCCGGTTTTATCAAAAGACAGCTGGTGGAGACACGCCAAATCACCAAACACGTTGCCCAGATCCTGGATTCGAGGATGAACACGAAGTATGACGAGAACGACAAGTTGATTAGGGAAGTCAAGGTCATCACTTTGAAGTCCAAGCTGGTGAGCGACTTTCGCAAAGACTTCCAGTTTTACAAAGTCAGGGAAATTAATAACTACCACCACGCCCACGACGCCTACCTTAACGCCGTGGTTGGCACAGCACTCATCAAGAAATACCCTAAGCTCGAATCTGAGTTCGTCTATGGCGACTATAAGGTCTACGACGTTAGAAAAATGATCGCGAAATCTGAGCAGGAAATAGGCAAGGCAACTGCCAAGTACTTCTTCTATTCCAATATCATGAACTTTTTTAAGACGGAGATTACCCTGGCGAATGGTGAGATCCGCAAGCGCCCTTTGATTGAGACAAACGGAGAAACAGGAGAGATCGTATGGGACAAAGGGCGGGACTTTGCTACTGTTAGGAAGGTGCTCTCTATGCCACAAGTTAACATTGTCAAAAAAACTGAAGTGCAGACAGGTGGGTTTAGCAAGGAATCTATCCTGCCGAAGAGGAACTCTGACAAGCTGATCGCCCGCAAGAAAGATTGGGATCCGAAAAAGTACGGAGGATTCGACTCCCCCACAGTTGCGTACTCCGTGCTTGTCGTGGCCAAAGTGGAGAAGGGCAAGTCTAAGAAGCTCAAGAGCGTCAAAGAGTTGTTGGGGATCACGATTATGGAGCGGTCGTCTTTCGAAAAGAATCCGATAGATTTTCTCGAGGCCAAGGGTTATAAAGAAGTCAAGAAGGATCTTATCATCAAGCTCCCTAAGTACTCCCTCTTTGAGCTTGAAAACGGACGGAAAAGAATGCTGGCTTCAGCGGGTGAACTTCAGAAGGGTAATGAACTCGCTCTGCCCTCAAAATATGTGAATTTCCTTTACCTGGCATCACACTATGAGAAGCTTAAGGGGTCTCCAGAGGACAACGAGCAGAAGCAACTGTTCGTTGAACAACACAAGCACTACCTTGACGAGATTATCGAGCAAATCAGCGAGTTTAGCAAGCGCGTTATACTGGCAGACGCAAATCTTGATAAGGTCCTTAGCGCCTACAACAAGCATAGAGACAAACCCATCCGGGAGCAGGCCGAGAACATTATTCATCTCTTCACCTTGACGAATCTTGGGGCCCCGGCCGCGTTCAAGTACTTCGATACTACCATAGACAGAAAGCGCTATACATCGACAAAGGAAGTTCTTGACGCCACGCTGATCCACCAAAGTATAACAGGCCTCTATGAGACACGCATCGACCTTTCGCAGTTGGGCGGTGACCGCCCCAAAAAGAAGAGGAAAGTTGGCGGGTGA
